# Giant Room‐Temperature Power Factor in *p*‐Type Thermoelectric SnSe under High Pressure

**DOI:** 10.1002/advs.202103720

**Published:** 2022-02-20

**Authors:** Natalia V. Morozova, Igor V. Korobeynikov, Nobuyoshi Miyajima, Sergey V. Ovsyannikov

**Affiliations:** ^1^ M. N. Miheev Institute of Metal Physics of Ural Branch of Russian Academy of Sciences 18 S. Kovalevskaya Str. Yekaterinburg 620137 Russia; ^2^ Bayerisches Geoinstitut Universität Bayreuth Universitätsstrasse 30 Bayreuth D‐95447 Germany; ^3^ Institute for Solid State Chemistry of Ural Branch of Russian Academy of Sciences 91 Pervomayskaya Str. Yekaterinburg 620219 Russia

**Keywords:** electrical resistivity, high pressure, SnSe, transmission electron microscopy, thermoelectric power

## Abstract

Materials that can efficiently convert heat into electricity are widely utilized in energy conversion technologies. The existing thermoelectrics demonstrate rather limited performance characteristics at room temperature, and hence, alternative materials and approaches are very much in demand. Here, it is experimentally shown that manipulating an applied stress can greatly improve a thermoelectric power factor of layered *p*‐type SnSe single crystals up to ≈180 µW K^−2^ cm^−1^ at room temperature. This giant enhancement is explained by a synergetic effect of three factors, such as: band‐gap narrowing, Lifshitz transition, and strong sample deformation. Under applied pressure above 1 GPa, the SnSe crystals become more ductile, which can be related to changes in the prevailing chemical bonding type inside the layers, from covalent toward metavalent. Thus, the SnSe single crystals transform into a highly unconventional crystalline state in which their layered crystal stacking is largely preserved, while the layers themselves are strongly deformed. This results in a dramatic narrowing in a band gap, from *E*_g_ = 0.83 to 0.50 eV (at ambient conditions). Thus, the work demonstrates a novel strategy of improving the performance parameters of chalcogenide thermoelectrics via tuning their chemical bonding type, stimulating a sample deformation and a band‐structure reconstruction.

## Graphical abstract

In the example of *p*‐type SnSe, a novel strategy of improving the thermoelectric performance of chalcogenide thermoelectrics via tuning their chemical bonding type, is demonstrated. Under applied high pressure, the SnSe crystals are severe deformed and their band structure is dramatically modified. This results in a colossal enhancement of their thermoelectric power factors at room temperature.

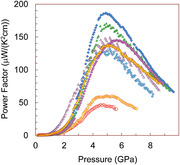

## 1 Introduction

High‐performance thermoelectric materials that can effectively convert heat into electricity, and vice versa, are very much demanded in various energy‐related applications, for example, in energy‐saving and refrigeration technologies. This circumstance explains enormous interest in fabrication and investigation of novel potential thermoelectrics. Besides, alternative methods and strategies that would allow to improve performance parameters of thermoelectrics, are also explored. The main parameters, which characterize performance of thermoelectrics are the power factor, PF = *S* ^2^ *σ* and the figure of merit, *ZT* = *TS* ^2^ *σ*/*λ* (where, *T* is the temperature, *S* is Seebeck coefficient (thermopower), *σ* and *λ* are electrical and thermal conductivities, respectively).^[^ ^1^, ^2^, ^3^, ^4^, ^5^, ^6^ ^]^ For several decades, Bi_2_Te_3_ and its‐based alloys, (Bi,Sb)_2_(Te,Se,S)_3_ were considered as the best thermoelectrics for near‐room‐temperature applications.^[^ ^7^ ^]^ Recent studies identified the number of promising alternative thermoelectrics, which could be also effectively utilized at room temperatures, for example, *n*‐type Mg_3_Sb,^[^ ^8^, ^9^, ^10^ ^]^ AgCuTe,^[^ ^11^ ^]^ MgAgSb,^[^ ^12^, ^13^ ^]^ nanostructured monoclinic Cu_2_Se,^[^ ^14^ ^]^ *n*‐type Mg_3_Bi_2_‐based materials,^[^ ^15^ ^]^ and some others. These facts inspire further search for more efficient room‐temperature thermoelectrics.

**Figure 1:**
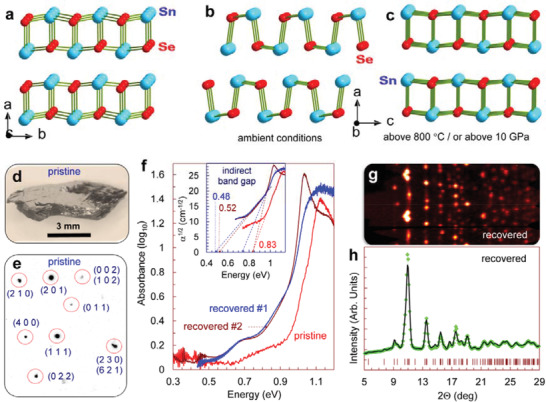
Structural and optical properties of SnSe crystals at 295 K. a–c) Layered crystal structure of SnSe in different crystallographic projections. Projection along the layers shown in (a), is the same for the ambient‐pressure *Pnma*, the high‐temperature *Cmcm*, and the high‐pressure *Bbmm* structures of SnSe. Whereas, the perpendicular projection along the layers in the ambient‐pressure *Pnma* structure (b) differs from projections in both the high‐temperature *Cmcm* and the high‐pressure *Bbmm* structures (c). d) Photograph of the investigated SnSe crystal. e) Section of X‐ray diffraction pattern of the SnSe crystal at ambient conditions. It is indexed in the orthorhombic *Pnma* structure. f) Near‐infrared absorption spectra of the pristine SnSe crystal and two samples recovered after the high‐pressure experiments. The inset shows the determination of the indirect band gaps in these samples. g) Azimuth X‐ray diffraction pattern of one of the samples recovered after the high‐pressure experiments. h) 2D X‐ray diffraction pattern obtained by integration of the azimuth pattern shown in (g). A Rietveld refinement of this pattern confirmed that this sample has the same *Pnma* structure as in the pristine crystal (e). The symbols are experimental data, the solid line is a calculated profile, and the dashes are anticipated reflections for *Pnma* symmetry.

Recently, the excellent thermoelectric properties of layered SnSe single crystals were discovered at high temperature above 800 K.^[^ ^16^ ^]^ This finding moved SnSe to the focus of intensive investigations.^[^ ^17^, ^18^, ^19^, ^20^, ^21^, ^22^, ^23^, ^24^, ^25^, ^26^, ^27^, ^28^, ^29^ ^]^ At ambient conditions, SnSe crystallizes in a layered structure with *Pnma* symmetry (**Figure** **1**a,b). It has an indirect band gap of about *E*_g_ ≈ 0.8–1 eV.^[^ ^16^, ^30^ ^]^ Above 800 K, the additional Sn‐Se bonds are formed in the crystal structure of SnSe along the *c*‐axis (Figure 1c), and the structure symmetry changes to higher‐symmetric *Cmcm*.^[^ ^16^ ^]^ The excellent thermoelectric properties of this *Cmcm* phase were explained by ultra‐low thermal conductivity, which was proposed to stem from strong anharmonicity of the chemical bonds.^[^ ^16^ ^]^ Remarkably, SnSe transforms to a similar crystal structure at room temperature, if subjected to applied high pressure above ≈10 GPa (Figure 1c).^[^ ^31^, ^32^ ^]^ It was predicted that this high‐pressure phase is also a high‐performance thermoelectric.^[^ ^33^ ^]^ Moreover, the number of studies experimentally established that even the ambient‐pressure *Pnma* phase can turn to a good room‐temperature thermoelectric, if appropriately doped.^[^ ^17^, ^18^, ^19^, ^34^, ^35^, ^36^, ^37^, ^38^, ^39^ ^]^

The energy gap of SnSe was predicted to dramatically decrease with pressure and to close before the phase transition at about 8–10 GPa.^[^ ^32^, ^40^ ^]^ Therefore, electronic properties of SnSe should be highly sensitive to applied stress. Kindred chalcogenides, for example, PbSe^[^ ^41^ ^]^ and SnTe,^[^ ^42^, ^43^ ^]^ also demonstrated strong pressure responses of their electronic properties.^[^ ^44^ ^]^ Recently, it was experimentally found that the power factor of both undoped and doped *p*‐type single crystals of SnSe is significantly improved upon hydrostatic pressurization up to 2 GPa.^[^ ^39^, ^45^ ^]^ This enhancement was explained by a multi‐valley conductivity resulting from a Lifshitz transition above 1 GPa.^[^ ^45^, ^46^ ^]^ Remarkably, Raman spectroscopic studies documented a dramatic softening of some phonon modes of SnSe with pressure above 1 GPa.^[^ ^47^ ^]^ This finding indicated an anharmonicity of the Sn‐Se bonds and suggested a possible change in the prevailing type of chemical bonding from covalent to metavalent.^[^ ^47^ ^]^ Recall, metavalent type of chemical bond was recently singled out for binary chalcogenides as “intermediate” between covalent bond (localized electrons) and metallic one (delocalized electrons);^[^ ^48^, ^49^, ^50^, ^51^ ^]^ and hence, it should be characterized by rather high electrical conductivity similar to metals, but with some degree of electron‐sharing similar to covalent materials.^[^ ^48^, ^49^, ^50^, ^51^, ^52^ ^]^ Therefore, one can surmise that applied stresses can cause intriguing changes in properties of SnSe, thus disclosing “chemical bonding–properties” relationship.^[^ ^51^ ^]^

**Figure 2:**
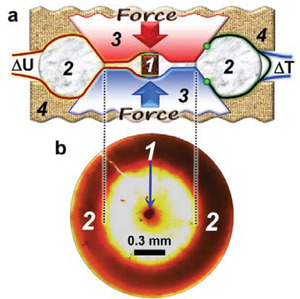
a) Simplified schematic side view of the cylindrical high‐pressure cell with hemispherical cavities in the anvils (1 – sample, 2 – toroidal‐shaped sample container made of limestone, 3 – anvils, 4 – supporting plungers). The bulk arrows labeled with “Force” indicate the direction of force application. The labels Δ*U* and Δ*T* denote the thermoelectric voltage and temperature difference outputs, respectively. b) A photograph of a limestone container (2) with a microscopic SnSe sample (1), recovered after the high‐pressure experiments.

In the present work, we measure the thermoelectric power and electrical resistivity of conventional undoped *p*‐type SnSe single crystals under cycling applied pressure up to 9 GPa at room temperature (**Figure** **2**a). We find that the power factor of the crystals is significantly improved under pressure of 5 GPa, and this effect is further enhanced after their deformation. We suggest a novel strategy for improving thermoelectricity in chalcogenides via tuning their chemical bonding, which can lead to sample deformation and band‐structure reconstruction.

## 2 Results and Discussion

**Figure 3:**
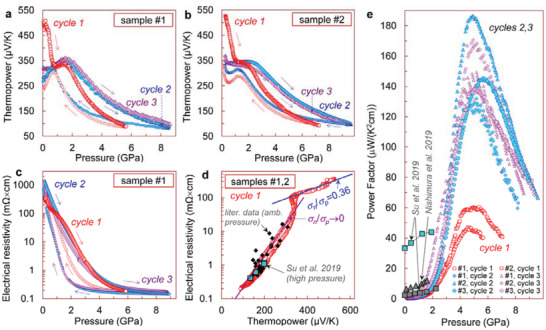
Thermoelectric properties of the SnSe crystals at 295 K. a,b) Pressure dependencies of the thermoelectric power for two samples, cut from the same SnSe crystal, for three successive pressurization and decompression cycles. The arrows indicate the directions of pressure variation. c) Pressure dependencies of the electrical resistivity of the SnSe crystal for three successive pressurization and decompression cycles. d) Parametric dependencies of the electrical resistivity versus the thermopower for the first pressurization cycle for samples #1 and #2. Using an approach described earlier,^[^ ^43^ ^]^ from the slopes of these curves the authors determined a ratio of partial electron and hole conductivities in the pristine crystal as *σ*_n_/*σ*_p_ = 0.36. For comparison, data from the literature data for *p*‐type SnSe single crystals, obtained at 300 K both at ambient pressure^[^ ^18^, ^19^, ^34^, ^35^, ^36^, ^37^, ^38^ ^]^ and at high pressure,^[^ ^39^ ^]^ is plotted here. e) Pressure dependencies of the thermoelectric power factor for three samples of the same SnSe crystal. For comparison, data from the literature for three different crystals, which are compressed in hydrostatic pressure conditions up to about 2 GPa,^[^ ^39^, ^45^ ^]^ are given.

We selected a high‐quality crystal of SnSe (Figure 1d) and verified its chemical composition and crystal structure. The crystal adopted the orthorhombic *Pnma* structure with the unit‐cell parameters as follows: *a* = 11.518 Å, *b* = 4.162 Å, *c* = 4.444 Å, and *V* = 213.06 Å^3^ (Figure 1a,b), in consistence with data from the literature.^[^ ^16^, ^17^, ^18^, ^19^, ^20^, ^21^, ^22^, ^30^, ^31^, ^32^ ^]^ At ambient conditions, the crystal had an indirect energy gap of *E*_g_ = 0.83 eV (Figure 1f) and a high electrical resistivity of *ρ* ≈ 0.38 Ω cm along the layers (*b*‐*c* plane in Figure 1a). These characteristics are typical for undoped *p*‐type SnSe crystals. To estimate the high‐pressure effect on the power factor of SnSe, we measured pressure dependencies of Seebeck coefficient and electrical resistivity up to 9 GPa for multiple pressure cycling for three single‐crystalline samples (labeled further as #1‐#3), cut from different parts of the selected crystal (Figure 1d,e). The results obtained for samples #1‐#3 were nearly identical, and in **Figure** **3** we give a representative dataset.

Both the Seebeck coefficient and the electrical resistivity of the SnSe samples were greatly decreased with pressure (Figure 3a–c). In addition, the thermopower curves for the first pressurization cycle demonstrated a pronounced step‐like feature (hump) at about 1–2 GPa. The resistivity curve for the first pressurization cycle exhibited an apparent convexity in this pressure range (Figure 3c). No clear signatures of the phase transition, which was reported in the literature above 8–10 GPa,^[^ ^31^, ^32^ ^]^ were observed in the curves. Remarkably, the pressure behavior of both the thermopower and the electrical resistivity for the second and third pressurization cycles significantly differed from their first cycles (Figure 3a–c). Earlier works also documented multi‐order drops in electrical resistivity of SnSe crystals in this pressure range, while, reported rather discrepant findings. In general, the pressure dependencies of the thermopower and the electrical resistivity for our SnSe samples can be explained by a significant narrowing in the semiconductor band gap with pressure, in line with predictions.^[^ ^40^ ^]^

The power factor of the SnSe samples was significantly enhanced with the applied pressure. At about 5 GPa, it achieved values of PF ≈ 45–60 µW cm^−1^ K^−2^ for the first pressurization cycle, and of PF ≈ 130–180 µW cm^−1^ K^−2^ for the following ones (Figure 3e). Band‐gap tuning is known to be an effective strategy to optimize the power factor of a semiconductor thermoelectric, since its electrical resistivity and thermopower depend on a band gap in different manners.^[^ ^44^ ^]^ In Figure 3d, we replotted the data for the first pressurization cycle in a parametric “resistivity versus thermopower” form. In the pressure range up to about 1 GPa, these data roughly corresponded to those for typical undoped *p*‐type single crystals, reported in the literature.^[^ ^16^, ^20^, ^35^, ^39^, ^45^ ^]^ Using an approach described earlier,^[^ ^43^ ^]^ from these “resistivity versus thermopower” curves, we estimated a ratio of partial electron and hole conductivities in the pristine crystal as *σ*_n_/*σ*_p_ ≈ 0.36 (Figure 3d). Above ≈2 GPa, our curves well coincided with data from the literature for *p*‐doped single crystals having hole concentrations in the range of 10^18^–10^20^ cm^−3^ (Figure 3d), obtained at room temperature both at ambient pressure,^[^ ^18^, ^34^, ^35^ ^]^ and at high pressure.^[^ ^39^ ^]^ Thus, the first pressurization cycle in our study, to some extent, corresponded to a charge‐carrier optimization via the band‐gap narrowing. Previous works found that the thermoelectric performance of SnSe is optimized at 300 K at hole concentrations of an order of 10^19^–10^20^ cm^−3^.^[^ ^53^ ^]^ The maximum power factor achieved in our study for the first pressurization cycle at 5 GPa amounted to about 45–60 µW cm^−1^ K^−2^ (Figure 3e). This value was approximately the same as the record values of ≈40–55 µW cm^−1^ K^−2^, reported to date for *p*‐doped SnSe crystals at 300 K.^[^ ^18^, ^36^, ^37^, ^38^, ^39^ ^]^ For the second and third pressurization cycles, the much higher values of PF ≈ 130–180 µW cm^−1^ K^−2^ were attained (Figure 3e).

Apparently, the step‐like feature (hump) in the thermopower curves at ≈1–2 GPa enhanced the Seebeck coefficients of the SnSe crystals (Figure 3a,b) and resulted in an additional improvement of their power factors. A recent study discovered an electronic Lifshitz transition in SnSe crystals above 1 GPa.^[^ ^45^ ^]^ Such transitions are accompanied by variations in band‐structure topology and can result in multi‐valley conductivity.^[^ ^45^, ^54^ ^]^ It was further established that in lightly‐doped *p*‐type crystals this transition completes at about 6 GPa and turns SnSe to a five‐valley semimetal.^[^ ^46^ ^]^ A multi‐valley character of electrical conductivity should enhance the effective mass of charge carriers and thereby maintain the high values of Seebeck coefficient.^[^ ^46^ ^]^ It was indeed experimentally observed for *p*‐type SnSe crystals above 1 GPa.^[^ ^45^ ^]^ Thus, the hump in our thermopower curves at 1–2 GPa (Figure 3a,b) can be attributed to this Lifshitz transition.^[^ ^45^, ^46^ ^]^ Note that the enhanced power factor of heavily *p*‐doped SnSe crystals attained at ambient conditions,^[^ ^18^, ^34^, ^36^, ^38^ ^]^ was also attributed in the literature to a multi‐valley conductivity. In the last case, the heavy *p*‐doping shifts the top of the valence band of SnSe, and for hole concentrations exceeding ≈4–5 × 10^19^ см^−3^ the second valence band should cross the Fermi level and contribute to electrical conductivity.^[^ ^18^, ^34^, ^55^ ^]^

Some previous high‐pressure investigations of SnSe also detected features at pressures corresponding to the above Lifshitz transition.^[^ ^45^, ^46^ ^]^ For example, an early Mössbauer spectroscopy study found changes in both isomer shift and quadrupole splitting in the pressure range of 1.4–3 GPa.^[^ ^56^ ^]^ Several Raman spectroscopy studies of SnSe documented a minor discontinuous drop in phonon frequencies at about 1–1.5 GPa,^[^ ^40^, ^47^, ^57^, ^58^, ^59^ ^]^ followed by a strong abnormal softening of $B_{3g}^{2}$ and $A_{g}^{3}$ phonon modes associated with in‐plane Sn—Se vibrations with pressure up to 10 GPa.^[^ ^47^ ^]^ This softening suggested an anharmonicity of the Sn—Se bonding, and it was linked to changes in chemical bonding from prevalently covalent to metavalent.^[^ ^47^ ^]^

To find out a reason of the irreversible changes in the pressurization curves of the Seebeck coefficient for our SnSe crystals after the first pressure cycle (Figure 3a,b), we examined their structural, optical, and microstructural properties at ambient conditions after the final decompression cycle. The X‐ray diffraction studies showed that the samples recovered after the pressure‐cycling experiments have the same *Pnma* structure and largely conserve a crystallographic orientation of the pristine single crystals (Figure 1g). Thus, the recovered SnSe samples were neither single‐crystalline nor polycrystalline. We analyzed their X‐ray diffraction patterns using a full‐profile Rietveld refinement (Figure 1h), although, peak intensities could not be accurate because of a very strong texture (Figure 1g). Typical parameters of their *Pnma* unit cell amounted to *a* = 11.502 Å, *b* = 4.160 Å, *c* = 4.449 Å, and *V* = 212.86 Å^3^ (Figure 1h), that is, only slightly differed from the initial magnitudes.

A near‐infrared absorption spectroscopy examination found that the recovered samples have an indirect band gap of about *E*_g_ ≈ 0.50 eV, that is, much smaller, compared to *E*_g_ = 0.83 eV in the pristine crystal (inset in Figure 1f). Although, traces of the original absorption edge were still well observable in the spectra of the recovered samples near 0.8 eV (inset in Figure 1f). Thus, the irreversible changes in properties of the samples after the first pressure cycle, in particular, the thermopower drop from ≈+550 down to ≈+350 µV K^−1^ (Figure 3a,b), should be related to the above irreversible reconstruction in the electronic band structure.

**Figure 4:**
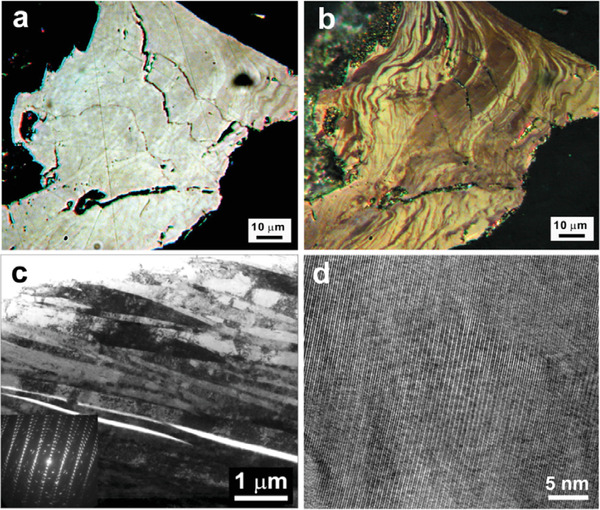
Microstructural characterization of the SnSe samples at 295 K. a,b) Optical images of the same well‐polished SnSe sample recovered from the high‐pressure experiments. They are obtained using a reflected polarized light microscope with a single nicol/polarizer (a) and with crossed nicols (b). Both images in different manners show smooth alterations in the crystallographic orientation throughout the sample. Apparently, these crystallographic variations can arise because of a severe plastic deformation inside the layers of the crystal (*b‐c* plane in Figure 1a). A more detailed optical image is given in Figure S6, Supporting Information. c) Bright‐field TEM image of the same recovered SnSe sample cut with a FIB across the layers (along the *a*‐axis). It demonstrates the formation of deformation bands at the submicron level resulted from combined interlayer glides. Selected area electron diffraction pattern is given as inset. d) Characteristic high‐resolution TEM (HRTEM) image of the same recovered SnSe sample. It shows the formation of numerous deformation lamellae and lattice fringes of the (*h*00) plane.

To figure out the origin of the above‐discussed band‐structure reconstruction, we investigated a microstructure of the SnSe samples recovered after the high‐pressure experiments both in visual examinations of their well‐polished surfaces using a polarized light optical microscope and by transmission electron microscopy (TEM) of their thin sections (**Figure** **4**). A surface morphology of the samples was highly unusual. It exhibited numerous wavy stripes of irregular shapes (Figure 4a.b). A more detailed optical image of these stripes is given in Figure S6, Supporting Information. These images unambiguously indicated a smooth but significant alteration in the crystallographic orientation throughout the sample. Apparently, these crystallographic variations could arise because of a severe plastic deformation inside the layers of the crystal (*b‐c* plane in Figure 1a). Using a focused ion beam (FIB), we cut this sample across the layers (shown in Figure S7, Supporting Information) and investigated its section by TEM. This examination revealed the formation of deformation bands at the submicron level (Figure 4c). This finding indicates that during the crystal deformation, the layers, which are bounded by weak van‐der‐Waals forces, tended to glide relative to each other without crystal breaking. Thus, one can conclude that the SnSe crystals were predominantly deformed inside the layers, while, they retained their layered stacking. In high‐resolution TEM studies, we discovered the formation of numerous deformation lamellae or bands and lattice fringes along the *a*‐direction (Figure 4d).

**Figure 5:**
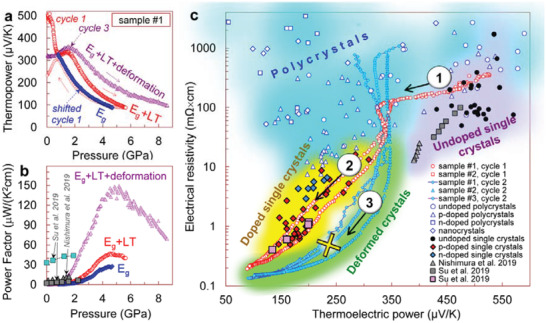
“Thermoelectric phase diagram” of SnSe at 295 K. a,b) Comparative pressure dependencies of the thermopower and the power factor for the SnSe crystals, replotted from Figure 3a,e for the first and third pressure cycles for sample #1. The arrows indicate the directions of pressure variation. The hump on the thermopower curves about 1–2 GPa (a) should correspond to the Lifshitz transition reported earlier.^[^ ^45^, ^46^ ^]^ The authors also plotted the first‐cycle pressurization curve with the subtracted hump, that is, shifted to the lower pressures. This curve, labeled as “shifted cycle 1”, demonstrates a hypothetical case, when the pressure behavior is determined by the band‐gap narrowing only. Near the curves in (a,b), factors that determined their pressure behavior are listed here.(“*E*_g_” – the band‐gap narrowing, LT – the Lifshitz transition, and the plastic deformation). The sample deformation mostly modified the contributions of both the band‐gap narrowing and the Lifshitz transition. For comparison, data from the literature,^[^ ^35^, ^39^ ^]^ are given in (b). c) “Thermoelectric phase diagram” of SnSe at 295 K in a form of “electrical resistivity versus thermopower” parametric dependencies for different sorts of samples. The authors plotted data from the present study as well as from the literature for polycrystals, undoped single crystals, and doped single crystals (see Supporting Information for more details, Figure S15 and Table S1, Supporting Information). In the present study, the authors started from the high‐quality undoped single crystals (1). The applied high pressure dramatically decreased the band gap and made them similar to *p*‐doped crystals (2). The pressure cycling enhanced their deformation and irreversibly converted them to “plastically‐deformed crystals” with the improved power factor (3).

The above TEM findings indicate that in the pressure range investigated the SnSe crystals were undergone a crossover from the original “brittle” to a more “ductile” state, facilitating their plastic deformation without cracking. This fact suggests that “viscous” forces dominated inside the layers of SnSe. After the applied high pressure was released, the crystals turned back from the “ductile” state to the “brittle” one, and many cracks appeared in their bodies due to high local strains (Figure 4a,b). The appearance of numerous cracks can explain a sizable increase in the electrical resistivity of the samples observed after the first decompression cycle (Figure 3c) toward magnitudes typical for polycrystals (**Figure** **5**c).

Uniform hydrostatic compression of samples should lead to their elastic deformation, and could hardly plastically deform them. Note, for example, that, even highly‐ductile metals compressed in hydrostatic pressure conditions, demonstrate elastic behavior, which makes it possible to determine their equation of states. Therefore, the deformation processes in the SnSe crystals were likely triggered by small non‐hydrostatic stresses across a sample, which often appear in solid pressure‐transmitting media because of their non‐zero shear moduli. For the toroidal geometry of the high‐pressure cell and the tiny sample sizes we used (Figure 2a,b), these potential non‐hydrostatic stresses across a sample should have been insignificant. But they seemed to be sufficient to drive a plastic deformation of the SnSe crystals. Likely, because of gradual grinding and re‐densification of a central area of a sample container under action of applied pressure, these small non‐hydrostatic stresses could be largely relaxed after the first pressure cycle. This could explain the absence of noticeable irreversible effects in the thermopower after the second and further pressure cycles (Figure 3a,b). In general, the SnSe samples showed no tendency to gradual deterioration and eventual amorphization with the pressure cycling. In case the SnSe crystals remained under pressure as brittle as at normal conditions, the small non‐hydrostatic stresses would not be capable to plastically deform them. The above can explain, for example, why in the previous studies in which the highly‐hydrostatic pressure conditions were used, a plastic deformation of SnSe crystals was not observed.^[^ ^31^, ^39^, ^45^, ^46^ ^]^

We presume that the crossover in mechanical properties of the SnSe crystals could become possible due to a drastic alteration in the nature of their chemical bonds under pressure. Recall that “brittle–plastic” transitions in chalcogenide solids are not so rare; for example, brittle Hg_1−_ *_x_* Cd*_x_* Se crystals demonstrated a plastic bending after treatment by high pressure up to 2 GPa.^[^ ^60^ ^]^ An earlier experimentally‐observed anomalous stretching of Sn‐Se bonds along the *c*‐axis in the layers (Figure 1b) with pressure,^[^ ^31^ ^]^ also hinted at possible change in the nature of these chemical bonds in SnSe. Since metavalent bonds are “intermediate” between “brittle” covalent and “plastic” metallic bonds,^[^ ^48^, ^49^, ^50^, ^51^, ^52^ ^]^ a tendency to ductility enhancement can be expected, when the prevailing type of chemical bonds inside the layers of SnSe alters from covalent to metavalent.

The pressure‐driven colossal enhancement of the power factor of the SnSe crystals (Figure 3e), should be contributed by three major factors, such as: i) the dramatic narrowing in the band gap, ii) the Lifshitz transition above 1 GPa,^[^ ^45^, ^46^ ^]^ and iii) the severe plastic deformation. However, individual contributions of these factors to the thermopower curves can hardly be accurately decoupled. Other factors, for example, the change in the chemical bonds, which also alter both the mechanical and electronic properties, are predominantly taken into account in the above three factors, and their influence on the thermopower via other band‐structure parameters (e.g., scattering parameter of carriers) seems to be insignificant. The pressure behavior of the Seebeck coefficient for the first pressurization cycle (Figure 3a,b) should correspond to the pristine single crystal, which has not yet been essentially deformed. Hence, these curves are mainly determined by a combined effect of the band‐gap narrowing and the Lifshitz transition (Figure 5a). Assuming that the hump in the thermopower curves above 1 GPa is a signature of the Lifshitz transition,^[^ ^45^, ^46^ ^]^ one can subtract this feature to single out an approximate contribution of the band‐gap narrowing to the thermopower curve (a blue curve example in Figure 5a). Neglecting a weak effect of the Lifshitz transition on the electrical resistivity of SnSe, a hypothetical power‐factor curve, corresponding to the band‐gap narrowing only, can be calculated too (Figure 5b). Comparing the thermopower and power–factor curves before and after this subtraction, one can get an idea of the magnitude of the Lifshitz transition contribution (Figure 5a,b).

For the second and third pressure cycles, the crystal deformation factor should be important. As found in our TEM examinations, while, the pristine layered crystal stacking of the SnSe crystals is largely preserved after their deformation, the layers themselves are plastically deformed (Figure 4). Hence, the Lifshitz transition in the deformed samples might be generally retained but significantly modified. From our thermopower data, one can figure out that in the deformed crystals the Lifshitz transition started at similar pressures but led to the higher Seebeck coefficients (cycles 2 and 3 in Figure 3a,b), compared to the pristine crystals (cycle 1). The sample deformation mostly modified the contributions of both the band‐gap narrowing (since the gap itself changed from 0.83 to 0.5 eV, Figure 1f) and the Lifshitz transition. The latter was decisive, and it provided the significant enhancement of the Seebeck coefficient (Figure 5a). Meanwhile, an influence of the plastic deformation on the Seebeck coefficient via variations in other band‐structure parameters (e.g., the ratio of partial hole and electron conductivities, scattering parameters and effective masses of charge carriers, etc.) seems to be incomparably less.

Thus, we showed that, starting from a conventional undoped SnSe crystal (label 1 in Figure 5c), one can firstly optimize its hole concentration via a controlled narrowing of the energy gap (label 2 in Figure 5c), and then, further enhance its Seebeck coefficient by a combined effect of the Lifshitz transition and plastic deformation (label 3 in Figure 5c). Our findings suggest that a more comprehensive theoretical analysis of the band structure and electronic properties of SnSe under high pressure in the Lifshitz transition region, is required to assess its high‐pressure thermoelectric potential. Although, SnSe was investigated in the number of theoretical works,^[^ ^58^, ^61^, ^62^, ^63^ ^]^ only recent studies identified the Lifshitz transition in *p*‐type SnSe^[^ ^45^ ^]^ and showed its sensitivity to hole concentration.^[^ ^46^ ^]^ As follows from our findings, such factors as: alteration in type of chemical bonds and plastic deformation should be taken into account.

We could not measure the thermal conductivity of our microscopic SnSe crystals under high pressure. But its electron (*λ*_e_) and lattice (*λ*_lat_) contributions can be approximately estimated. The former can be reasonably well determined using the Weidemann‐Franz Law as follows: *λ*_e_ = *LTσ*, where *L* is the Lorentz number, *T* is the temperature, and *σ* is the electrical conductivity.^[^ ^64^ ^]^ Given the Lorenz number of *L* ≈ 1.4 × 10^−8^ W Ω K^−2^ determined for Seebeck coefficients of an order of ≈180–220 µV K^−1[^ ^64^ ^]^ and the electrical resistivity of ≈0.5 mΩ cm at ≈4 GPa, we find *λ*_e_ ≈ 0.84 W K^−1^ m^−1^ at 4 GPa. Lattice thermal conductivity of layered SnSe single crystals depends on crystallographic direction, and it was reported in the literature to vary in the range of *λ*_lat_ ≈ 0.5–1.9 W K^−1^ m^−1^ at 300 K.^[^ ^16^, ^65^ ^]^ For a rough estimation of the figure of merit, *ZT* at 295 K, we can use the maximum magnitude, *λ_lat_* ≈ 1.9 W K^−1^ m^−1^ and assume that its increase due to the sample densification is largely compensated by its decrease due to the sample deformation (Figure 4d). Hence, at 4 GPa for sample #2, we find *ZT* ≈ 0.7 for the first pressurization cycle, which roughly corresponded to the single‐crystalline state, and *ZT* ≈ 1.5 for the second cycle for already the plastically deformed sample.

There are not so many thermoelectrics with high power factors at room temperature.^[^ ^66^ ^]^ In Tables S1 and S2, Supporting Information, we summarize data from the literature for SnSe and some other thermoelectrics. The maximum value of PF ≈ 45–60 µW cm^−1^ K^−2^ attained for the first pressurization cycle at 5 GPa for our samples (Figure 3e) corresponded to the state‐of‐the‐art values of PF ≈ 40–55 µW cm^−1^ K^−2^, reported to date in the literature for *p*‐doped SnSe single crystals at 300 K (Table S1, Supporting Information).^[^ ^18^, ^36^, ^37^, ^38^, ^39^ ^]^ Among the other near‐room‐temperature thermoelectrics with significant power factors, one can mention, for instnce, (Bi_1−_ *_x_* Sb*_x_*)_2_Te_3_,^[^ ^7^, ^67^, ^68^ ^]^ *n*‐type Mg_3_(Sb,Bi)_2_,^[^ ^8^, ^9^, ^10^, ^15^ ^]^ MgAgSb,^[^ ^12^, ^13^, ^69^ ^]^ AgSbTe_2_,^[^ ^1^ ^]^ Ag_2_Se,^[^ ^70^ ^]^ and *n*‐type Bi‐doped PbTe (Table S2, Supporting Information).^[^ ^71^ ^]^ The maximum value of PF ≈ 130–180 µW cm^−1^ K^−2^ achieved in the plastically‐deformed SnSe crystals at ≈5 GPa (Figure 3e) is among the highest known to date for room temperatures. Comparable magnitudes were reported only for *p*‐type half‐Heusler Nb_1−_ *_x_* Ti*_x_* FeSb alloy,^[^ ^72^ ^]^ F4‐TCNQ‐doped FASnI_3_ thin films,^[^ ^73^ ^]^ and for FeSe ultrathin films (Table S2, Supporting Information).^[^ ^74^ ^]^

## 3 Conclusion

In summary, we have demonstrated that manipulating an applied stress of several GPa can stimulate a plastic deformation of a conventional SnSe crystal and greatly enhance its power factor at room temperature. The pressure‐induced enhancement of the power factor in SnSe up to about PF ≈ 40–55 µW cm^−1^ K^−2^ at about 4–5 GPa should be related to both the gradual band gap narrowing that optimizes a charge carrier concentration, and the Lifshitz transition above 1 GPa, which stabilizes high Seebeck coefficient values.^[^ ^45^ ^]^ We have revealed that a plastic deformation of SnSe crystals results in a strong enhancement of the thermopower in the region of the Lifshitz transition, compared to the pristine crystal. This led to a giant enhancement of the power factor up to values of ≈180 µW K^−2^ cm^−1^ at 4–5 GPa. These effects could be realized, for example, in thin deformed films with controlled internal strains.

## 4 Experimental Section

### Sample Characterization

Conventional commercially available bulk crystals of SnSe of 99.999% purity from Chempur company were used. Using X‐ray diffraction methods, a high‐quality bulk crystal was selected for the thermoelectric and other measurements (Figure 1d,e). The X‐ray diffraction examinations were accomplished on a high‐brilliance Rigaku diffractometer with a wavelength of *λ* = 0.5594 Å. They confirmed that the selected crystal has the *Pnma* structure with unit‐cell parameters as follows: *a* = 11.518 Å, *b* = 4.162 Å, *c* = 4.444 Å, and *V* = 213.06 Å^3^. These parameters were similar to those reported in previous works. For the pressure‐dependent measurements of the thermopower and the electrical resistivity the authors cut from this crystal and selected several microscopic single‐crystalline grains.

### Electrical and Optical Properties at Ambient Conditions

The electrical resistivity of the selected SnSe crystal was measured on a rectangular‐shaped sample by the conventional van der Pauw method. It was found that at ambient conditions the resistivity along the layers (*b‐c* plane) (Figure 1a,b) amounts to *ρ* = 0.38 Ω cm. To determine the band gap, both the pristine crystal and the samples recovered after the high‐pressure cycling experiments were examined by near‐infrared absorption spectroscopy. Absorption spectra were acquired in the range of 2000–10 000 cm^−1^ on a Bruker IFS 120 Fourier transform spectrometer. For these spectroscopic investigations, a piece of the pristine crystal and two recovered samples were polished from both sides to a thickness of about 20 µm.

### High‐Pressure Cycling Experiments

The measurements of the thermoelectric power and the electrical resistivity under high‐pressure cycling were accomplished at room temperature using an automated mini‐press setup.^[^ ^75^ ^]^ This setup allowed a gradual generation of a force applied to a high‐pressure cell with a sample and simultaneous recording all output signals. Values of the applied force were automatically measured directly on the high‐pressure cell by a digital dynamometer. Pressure values inside the cell were determined from calibration curves based on well‐known and distinctly observable phase transitions in different materials. The calibration curves were determined for multiple pressurization cycles to account for changes in geometrical parameters of highly‐compressible components, such as a sample container.^[^ ^76^ ^]^ The setup allowed to perform automatic measurements of all output signals under multiple pressurization and decompression cycles.

### Seebeck Coefficient and Electrical Resistivity under High Pressure

The samples of SnSe were compressed from ambient pressure up to about 9 GPa in an anvil‐type toroidal high‐pressure cell, loaded into the above‐discussed mini‐press setup. The anvils with a culet size of about 1 mm had hemispherical cavities in the center (Figure 2a). A toroidal‐shaped limestone container served both as a gasket and as a pressure‐transmitting medium.^[^ ^77^ ^]^ A sample with sizes of about 150 × 150 × 150 µm^3^ or less was loaded into a hole in the center of this container (Figure 2b). The toroidal geometry of the high‐pressure cell (Figure 2a) provided both a uniform pressure in the center of the container and a uniform supporting pressure inside the container around the anvils tips.^[^ ^78^, ^79^, ^80^ ^]^ Hence, it provided a quasi‐hydrostatic compression regime. Potential non‐hydrostatic stresses across a sample, which likely did not exceed ≈0.1–0.2 GPa at all pressures up to 9 GPa, could not noticeably influence the results of the measurements.

The measurements of the thermoelectric power and the electrical resistivity were carried out along the layers (*b*‐*c* plane) (Figure 1a). In the thermopower measurements, an upper anvil was heated up by an electrical heater to generate a temperature difference (Δ*T*) of several Kelvins along a thickness of a sample.^[^ ^81^ ^]^ This temperature difference, Δ*T* was measured by thermocouples attached to the anvils near the tips. The authors estimated a potential uncertainty in determination of the Δ*T* value to be less than 5%. A thermoelectric voltage, Δ*U* generated by this Δ*T* was measured by two electrical probes. The authors verified linear dependencies of the thermoelectric voltage on the temperature difference, Δ*U*(Δ*T*) (Figure S1, Supporting Information).

The electrical resistivity was measured by a quasi‐four‐probe method (two bifurcated probes). This method was found to be much more sustainable under multiple pressure cycling, than the traditional four‐probe method. For electrical resistivity values somewhat exceeding 50 *μ*Ω cm, this method was proven to give rather accurate results. For example, it was successfully utilized to measure a pressure dependence of electrical resistivity of SnTe crystal with *ρ* ≈ 130 *μ*Ω cm.^[^ ^43^ ^]^ The limestone pressure‐transmitting container used protected a sample from fracture and allowed only slight variations in its linear sizes. Thus, a plastic deformation of a sample under applied pressure could only insignificantly influence its thickness and surface area. The authors controlled changes in geometrical sizes of a sample after the experiments both in visual examinations and by thickness measurements, and took this point into account when calculating the electrical resistivity. This examination also confirmed the absence of any notable radial pressure components during the measurements, which would shift a sample from the center to side.

### Transmission Electron Microscopy (TEM)

TEM examination was performed using a Philips CM20 FEG (Field Emission Gun) and an FEI Titan G2 80‐200 S/TEM, equipped with an energy dispersive X‐ray analyzer, operated at 200 kV. The TEM foils were prepared at voltages of 4 kV and incidence angle of 8 degrees by the conventional Ar‐ion milling method. For a deformed striped sample (Figure 4), a specific site sampling of a TEM lamella was performed using a dual beam focused ion milling machine (FEI Scios).

## Conflict of Interest

The authors declare no conflict of interest.

## Supporting information

Supporting Information

## Data Availability

The data that support the findings of this study are available from the corresponding author upon reasonable request.
