# Supplementary material for: Giant Room‐Temperature Power Factor in p‐Type Thermoelectric SnSe under High Pressure
Source: Adv Sci (Weinh). 2022 Feb 20;9(20):2103720. doi: 10.1002/advs.202103720 (PMC9284162; doi:10.1002/advs.202103720)
Supplement: Supplementary file 1 — Supporting Information [file ADVS-9-2103720-s001.pdf]

ADVANCED SCIENCE
Open Access

Supporting Information

for *Adv. Sci.*, DOI 10.1002/advs.202103720

Giant Room-Temperature Power Factor in *p*-Type Thermoelectric SnSe under High Pressure

*Natalia V. Morozova, Igor V. Korobeynikov, Nobuyoshi Miyajima and Sergey V. Ovsyannikov**

Supporting Information

Giant Room-Temperature Power Factor in p-Type Thermoelectric SnSe under High Pressure

Natalia V. Morozova, Igor V. Korobeynikov, Nobuyoshi Miyajima, Sergey V. Ovsyannikov**

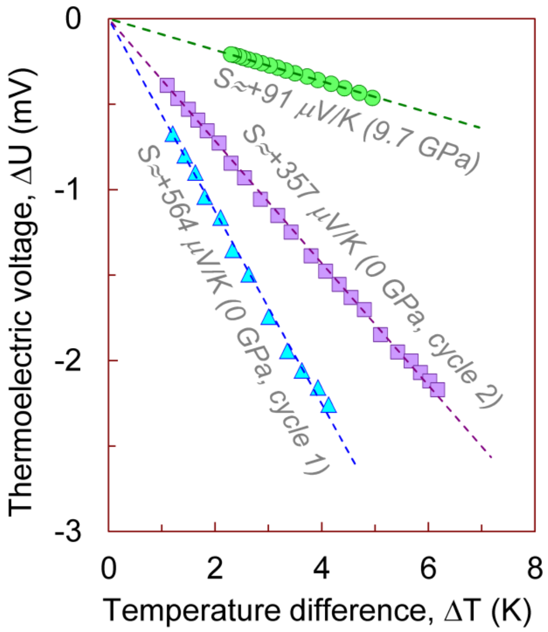

Figure S1. Dependencies of the thermoelectric voltage (ΔU) on the temperature difference (ΔT) across one of SnSe samples, measured at 295 K at P=0 GPa before the first and second pressure cycles, and at P=9.7 GPa, the maximal pressure achieved in the second cycle. In the settings used, the Seebeck coefficient (S) is determined as $S=-\Delta U/\Delta T$. These values are given near the curves.

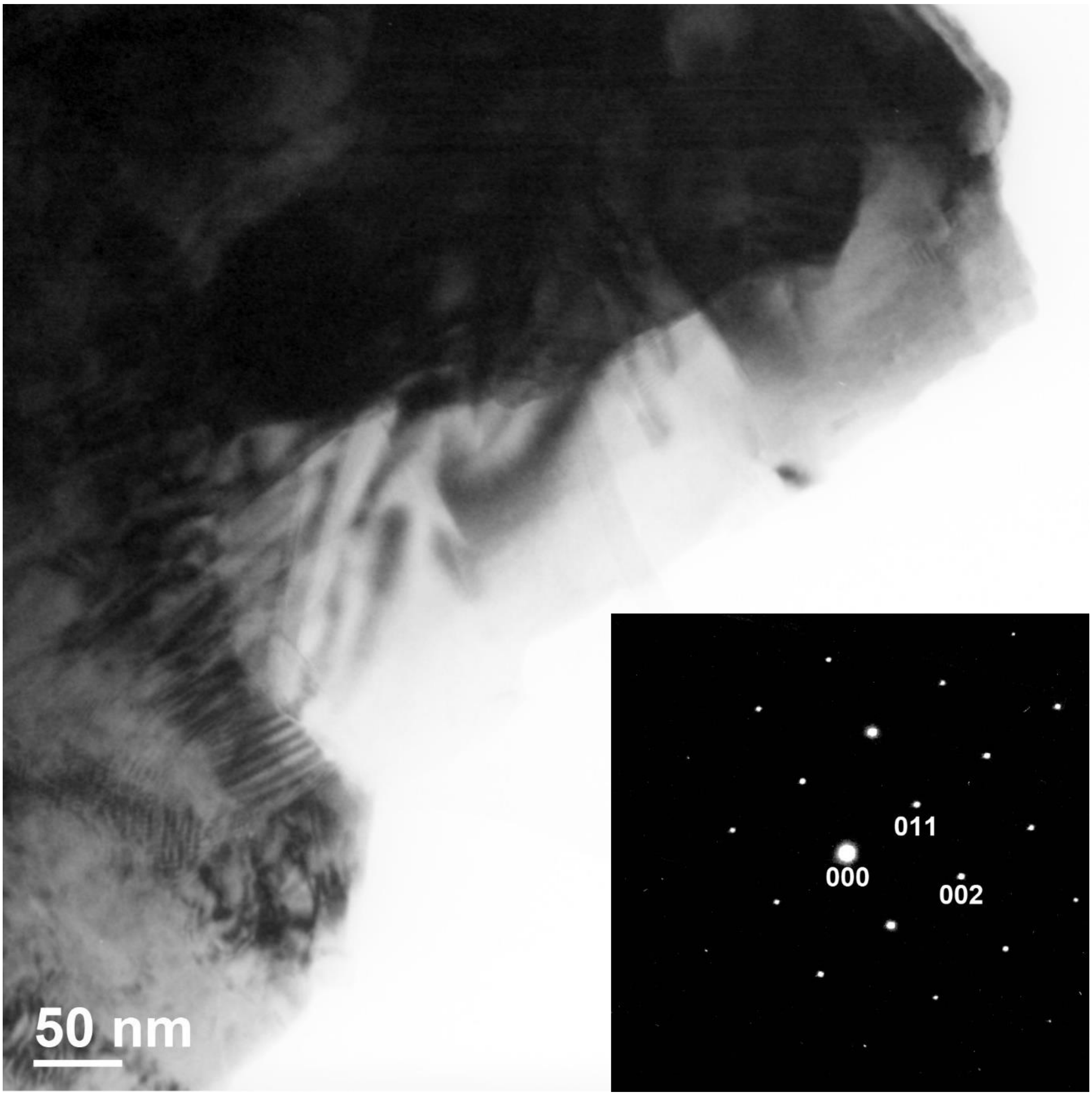

Figure S2. Bright-field TEM image of the *b*-*c* plane of one of the SnSe samples recovered from the high-pressure experiments. The inset shows its selected area electron diffraction pattern of the [100] zone axis. The upper right edge is the area of the HRTEM image (Figure S3).

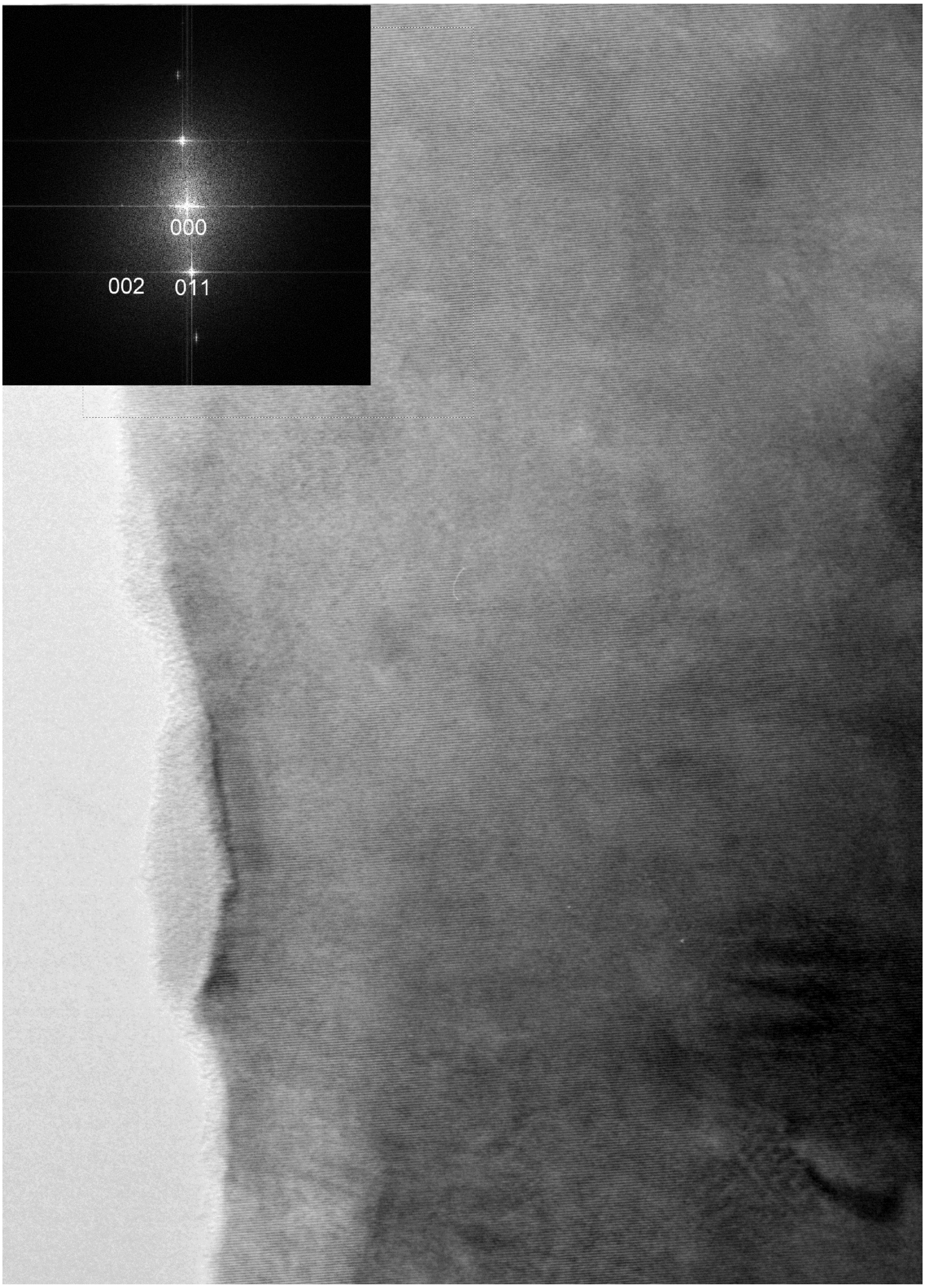

Figure S3. High-resolution transmission electron microscopy (HRTEM) image of the recovered SnSe sample from Figure S2. The inset is the electron diffraction pattern of the [100] zone axis obtained by using fast Fourier transformation (FFT) of the upper left area in this HRTEM image.

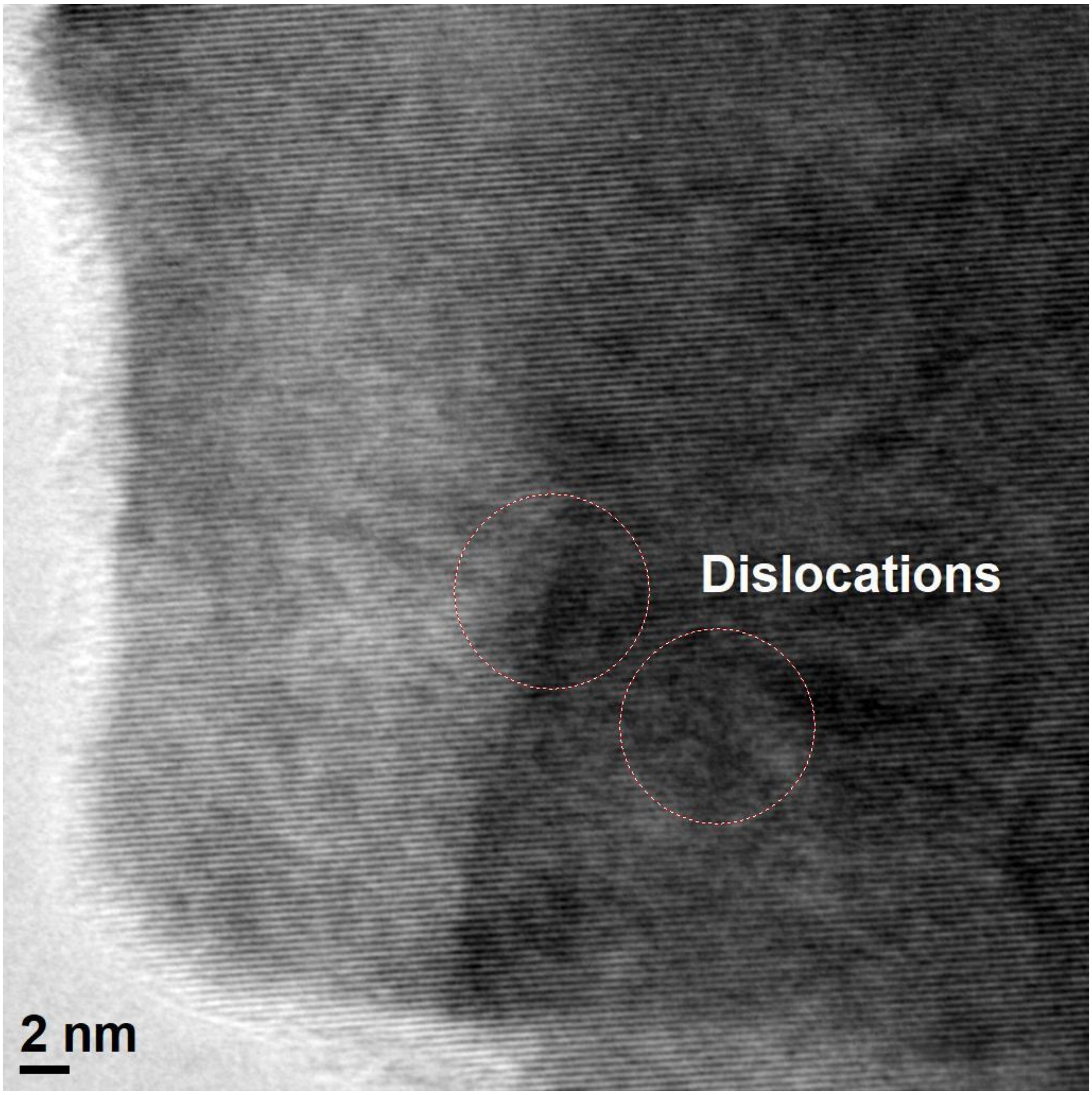

Figure S4. High-resolution lattice image of the recovered SnSe sample from Figure S2. Two examples of dislocations are highlighted by dashed circles.

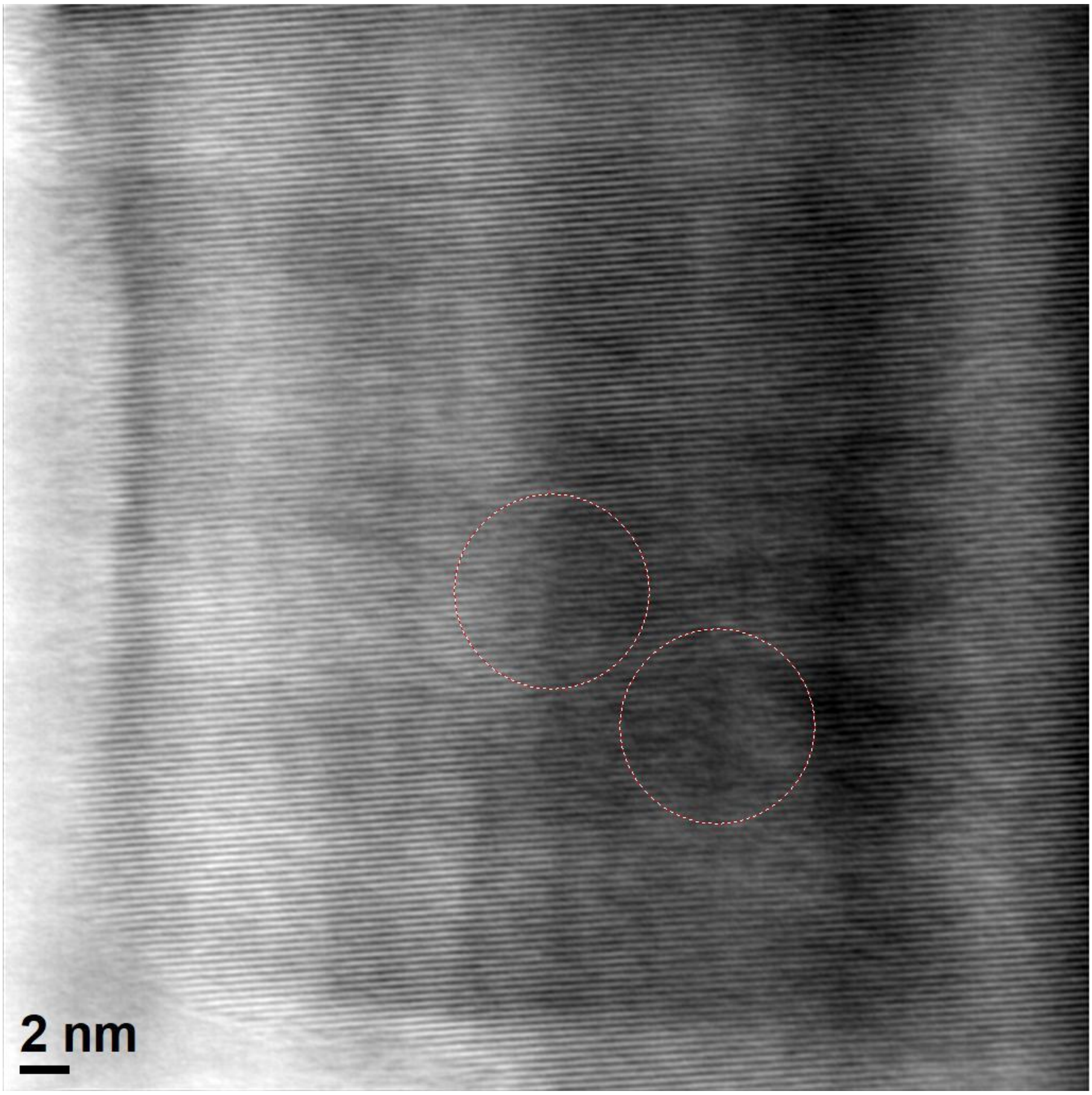

Figure S5. Wiener-filtered HRTEM image of Figure S4. The rotational averaging prosses of the Fast Fourier Transformation (FFT) of the HRTEM image was performed by a plugin-software based on procedures reported in Ref. S1.

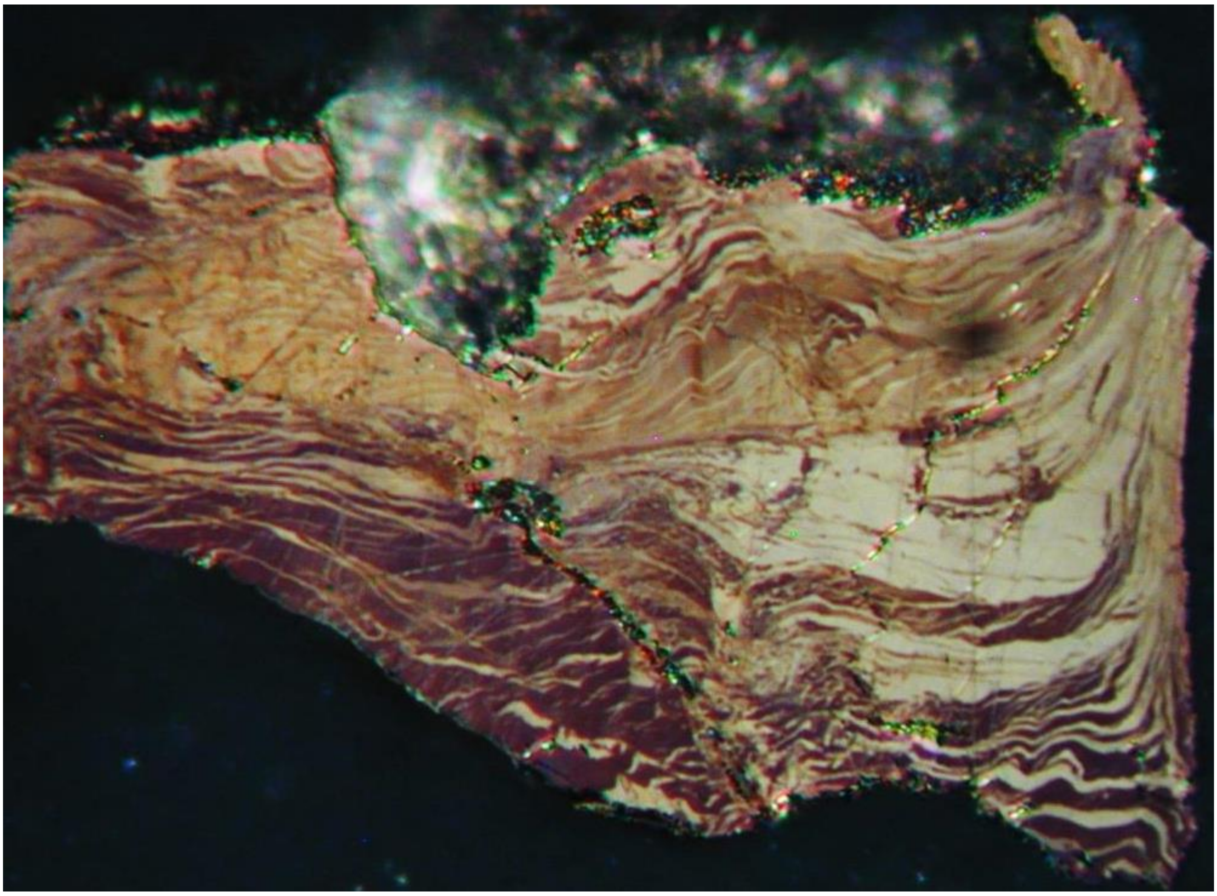

Figure S6. Reflective polarized light microscope image (with cross-polarizers) of a well-polished part of one of the SnSe sample recovered from the high-pressure experiments. The surface morphology indicates the variation of the crystallographic orientation of the crystal after its severe plastic deformation under high pressure.

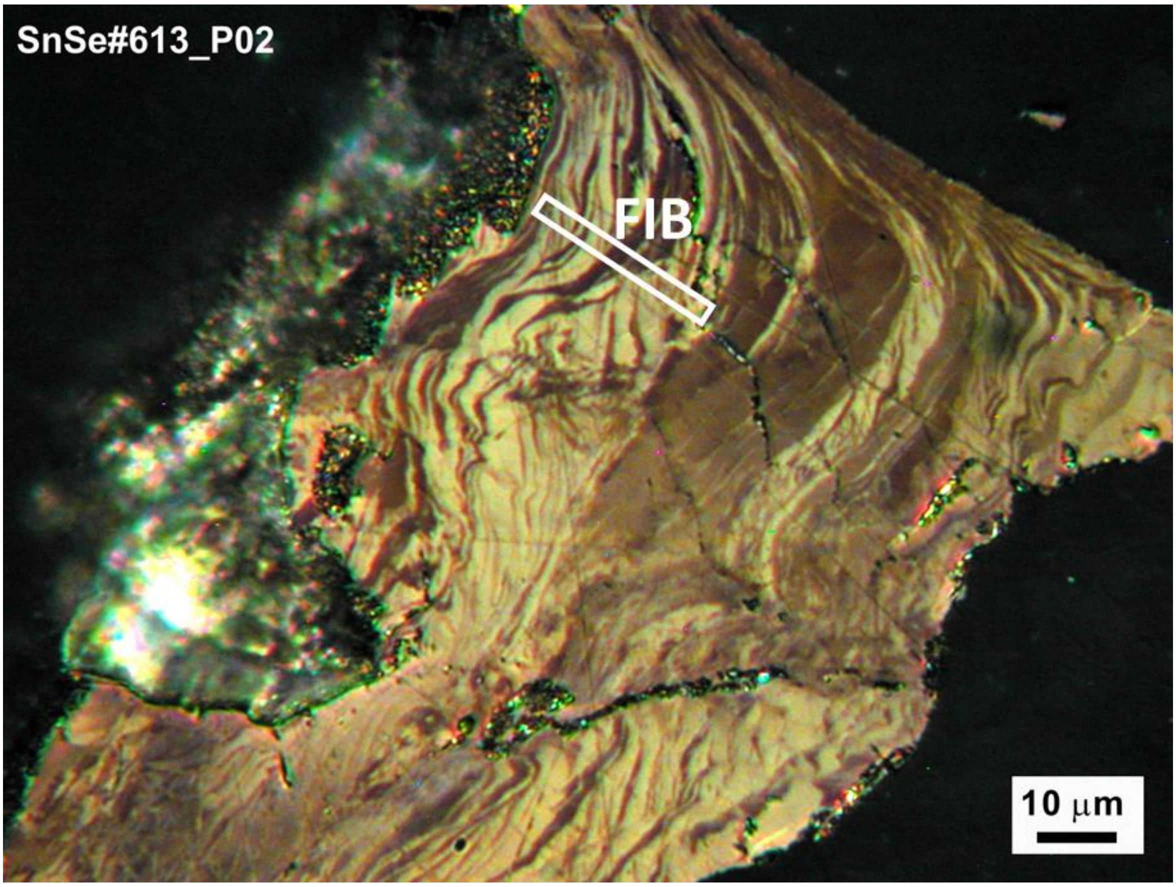

Figure S7. Reflective polarized light microscope image of the site of FIB-microsampling of the SnSe sample, shown above in Figure S6.

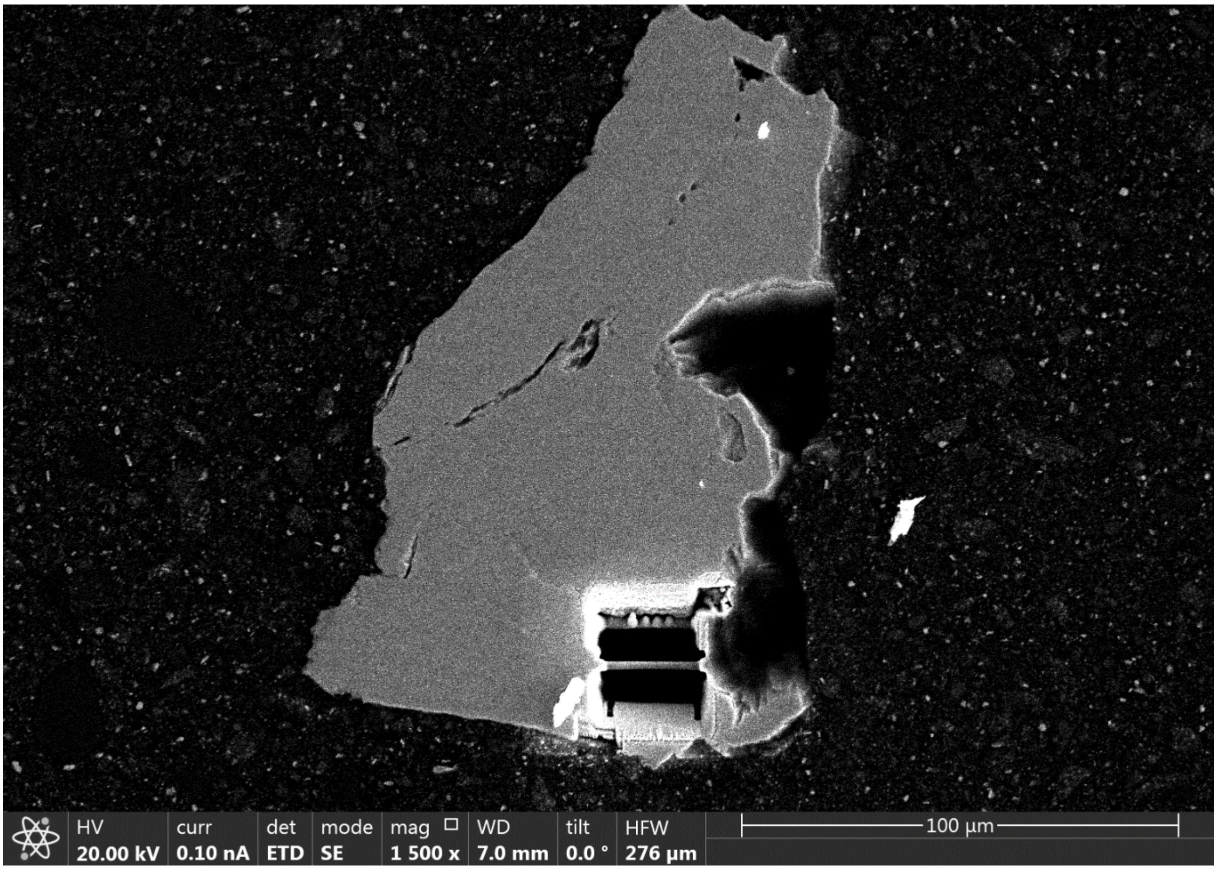

Figure S8. The SEM image of the recovered SnSe sample after the FIB-microsampling (Figure S7).

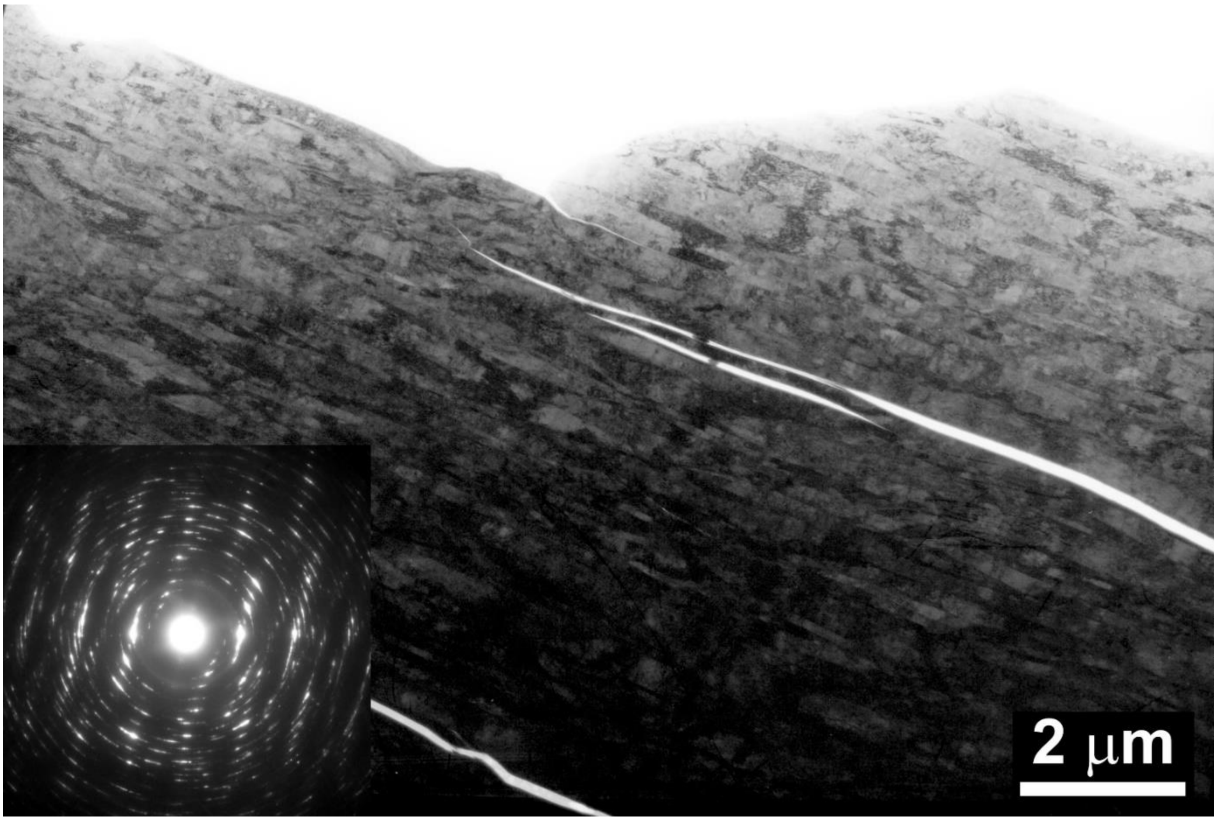

Figure S9. Bright-filed TEM image of the recovered SnSe sample shown in Figure S8 and which was cut across the layers. It demonstrates a set of deformation bands. Selected area electron diffraction (SAED) pattern is given as inset.

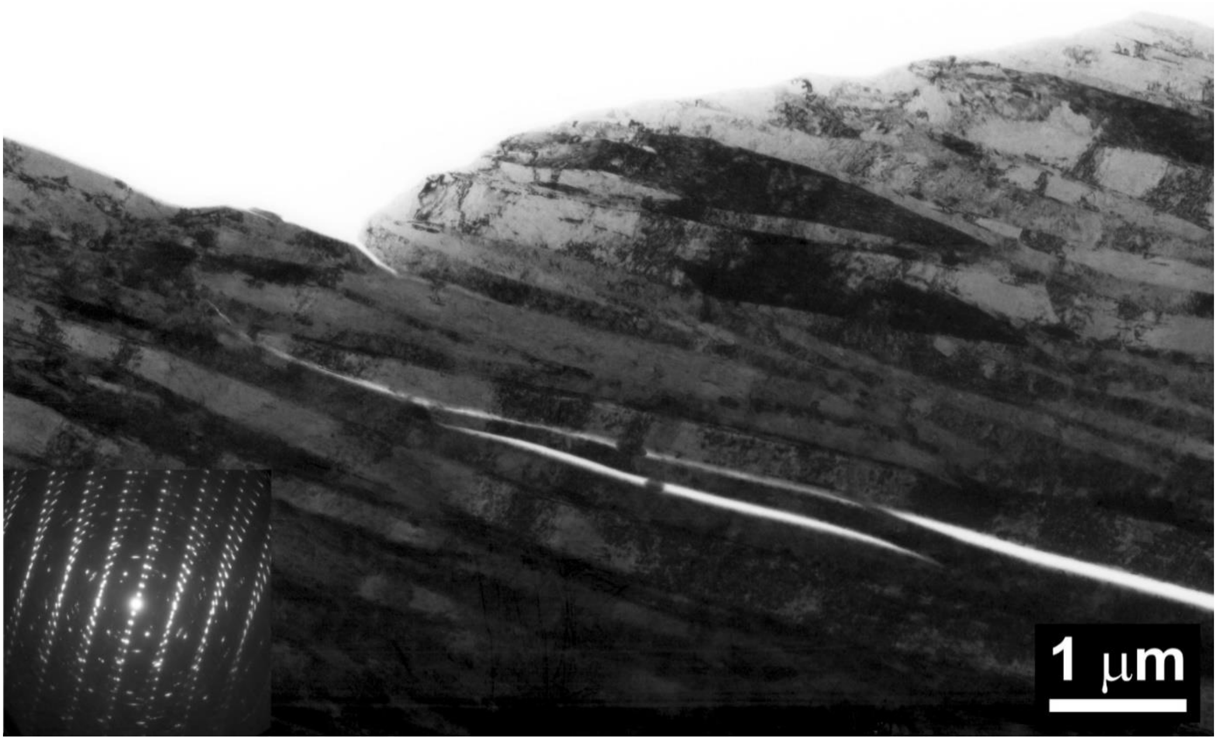

Figure S10. A magnified upper part of the bright-filed TEM image of the recovered SnSe sample shown in Figure S9. This plot better illustrates parameters of the deformation bands formed. The SAED pattern is given as inset.

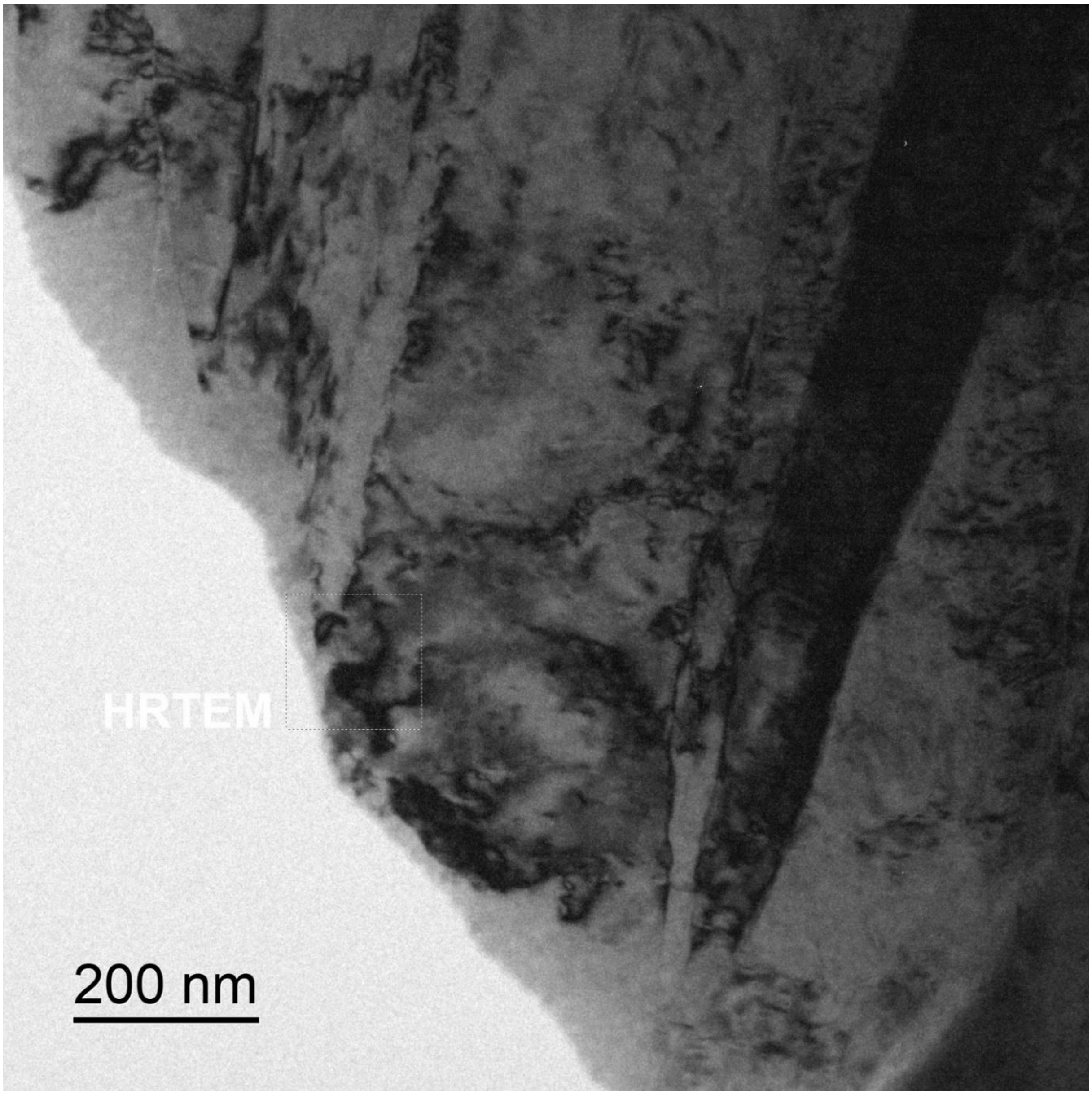

Figure S11. Bright-filed TEM image of one of the recovered SnSe sample from the high-pressure experiments. The dashed rectangular shows an area selected for high-resolution transmission electron microscopy (HRTEM) images (Figure S12, S13).

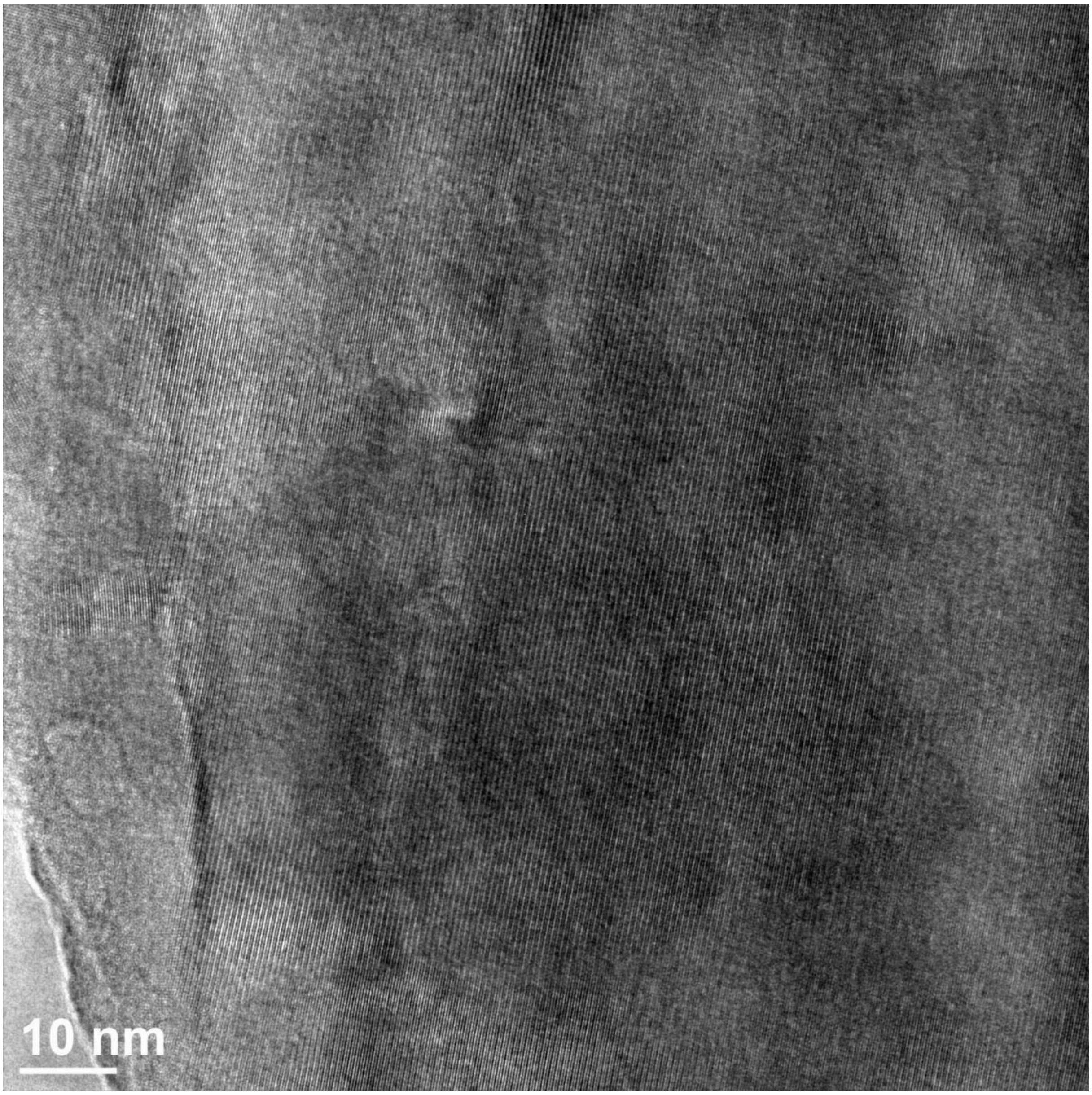

Figure S12. Wiener-filtered HRTEM image of the recovered SnSe sample taken in the area highlighted in Figure S11. One can see some contrasts with different frequencies of lattice fringes along the *a*-direction.

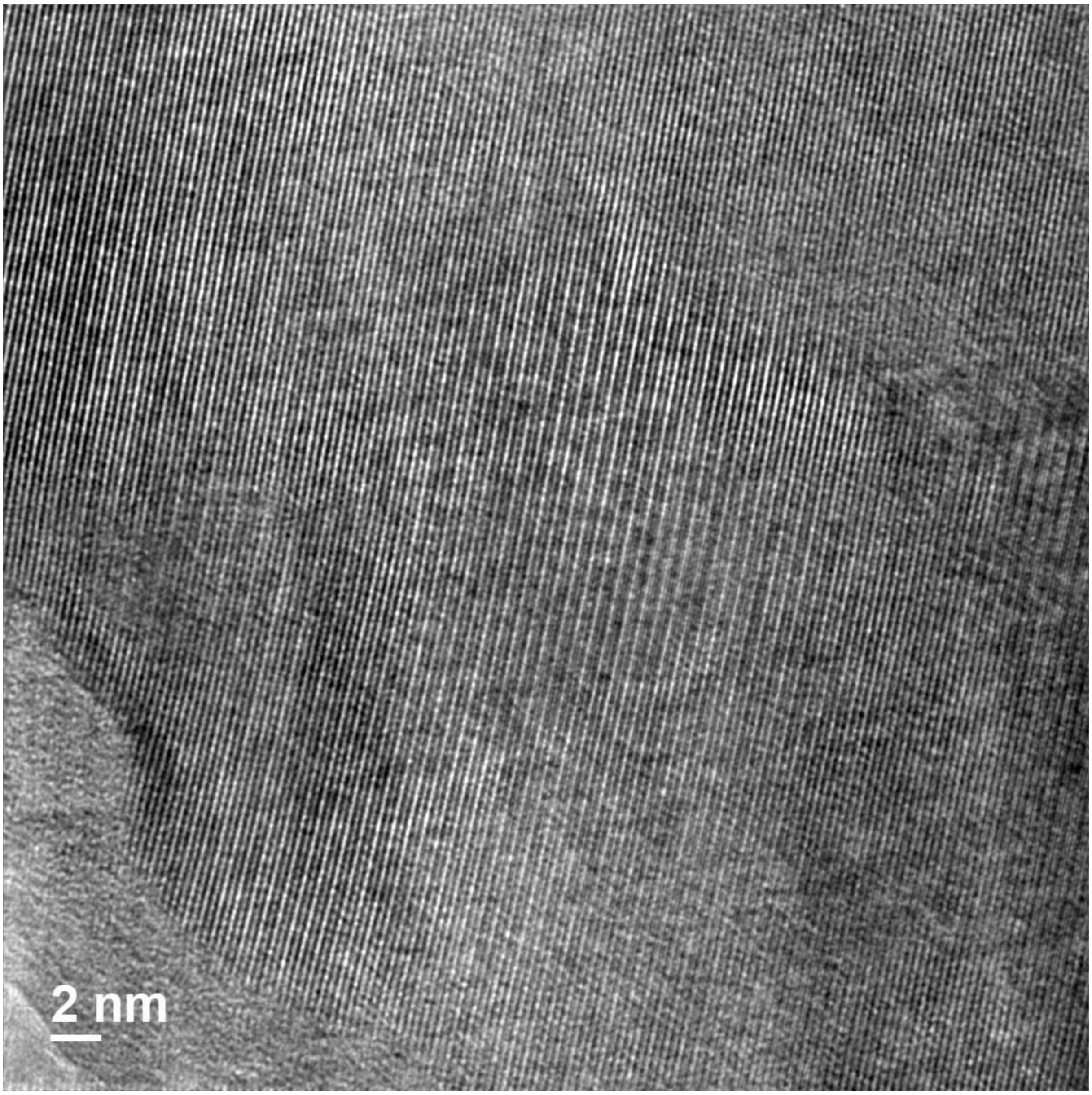

Figure S13. Wiener-filtered HRTEM image of the recovered SnSe sample taken in the area highlighted in Figure S11. One can see numerous deformation lamellas and lattice fringes along the *a*-direction.

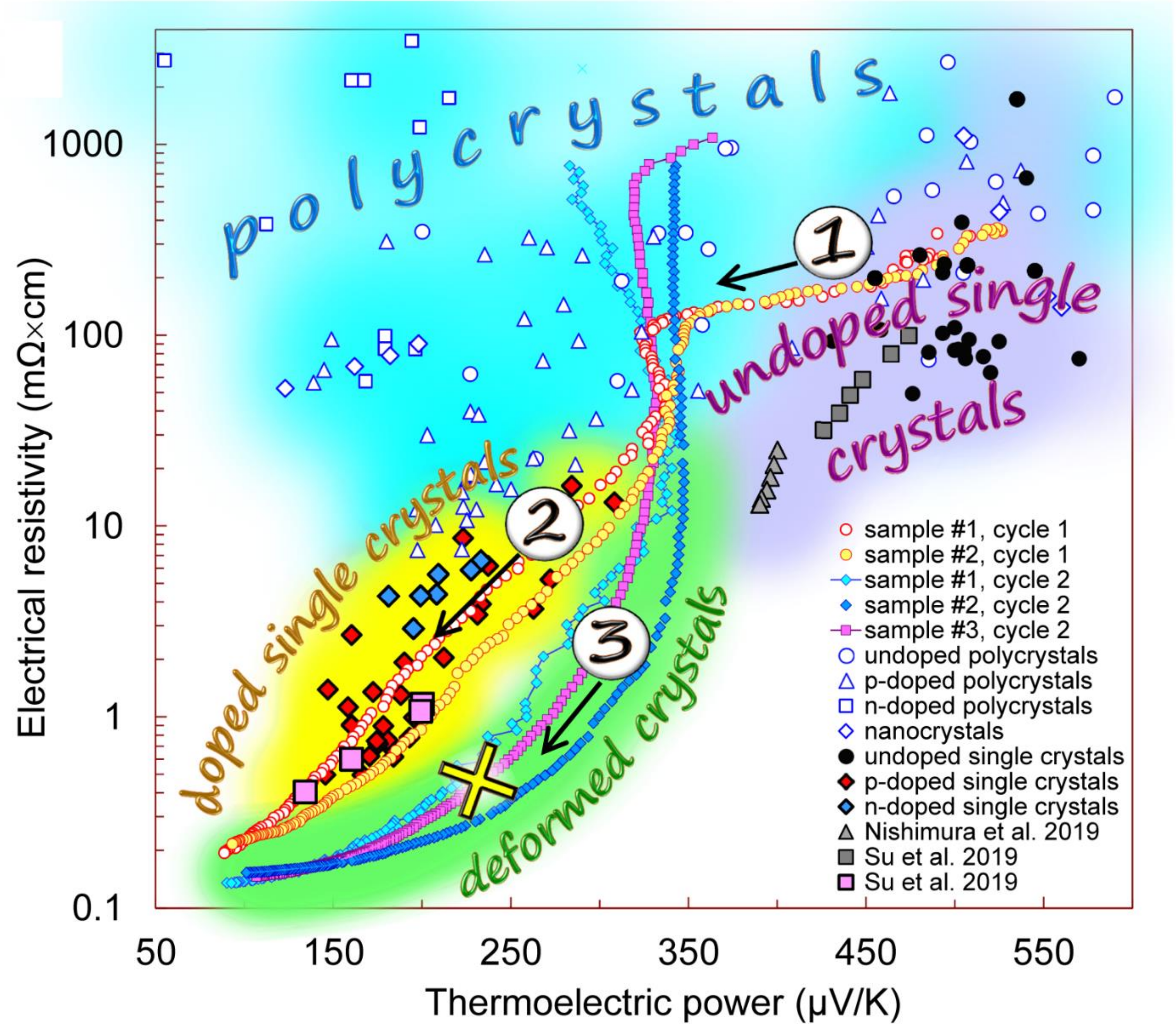

Figure S14. 'Thermoelectric phase diagram' of SnSe in the form of 'electrical resistivity *vs* thermopower' parametric dependencies at 295 K for different sorts of samples (polycrystals, undoped single crystals, *p*-doped single crystals, deformed crystals) combining our and the literature data. More details of this diagram, including all the literature references used, are given in Figure S15 and in Table S1, below. In our study, we started from high-quality undoped single crystals of SnSe (1). With the pressure application, they firstly turned to *p*-doped crystals because of the strong band-gap decreasing, combined with the Lifshitz transition, and then, to plastically deformed crystals (3).

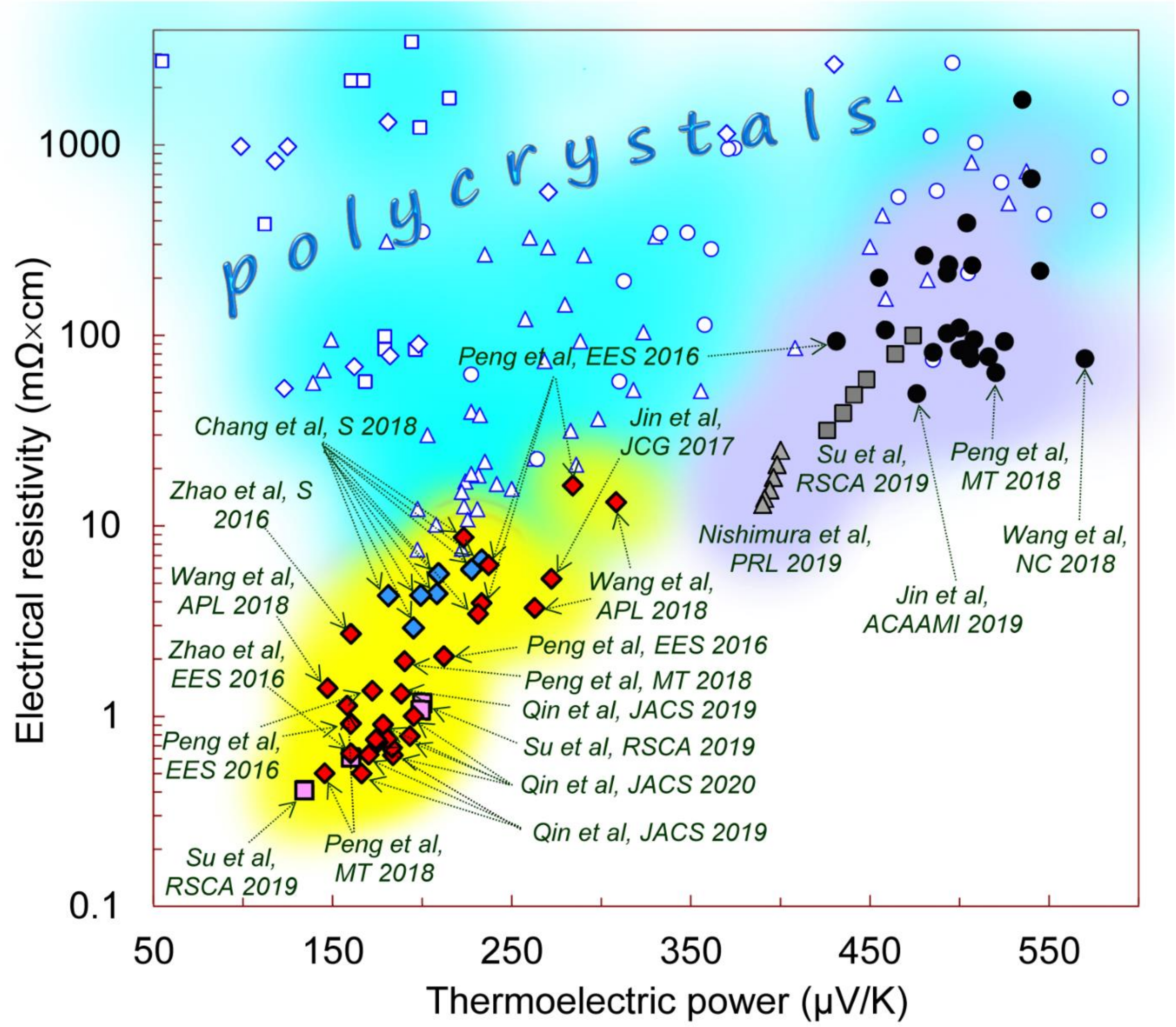

Figure S15. Selected literature data used in Figure S14. More details are given in Table S1, below.

Table S1. Electronic and thermoelectric properties of different SnSe samples at 300 K, summarized from the literature.

**Undoped SnSe single crystals**								
carrier concentration (10^{17} cm^{-3})	carrier mobility (cm^2 V^{-1} s^{-1})	Thermoelectric power (μV K^{-1})	Electrical conductivity (S cm^{-1})	Electrical resistivity (mΩ cm)	Power factor (μW K^{-2} cm^{-1})	Lattice thermal conductivity (W K^{-1} m^{-1})	along axis	Reference
3.1	30.7	540	1.5	666.7	0.44	0.46	*a*	Zhao *et al*, N 2014 (Ref. S2)
2.7	243.5	508	10.5	95.2	2.7	0.7	*b*	
5.9	131	516	12.9	77.5	3.4	0.67	*c*	
4.2	138.2	458.4		107	1.96	1.78	*b*	Wang *et al*, APL 2018 (Ref. S3)
4.45	150.3	431	10.7	93.5	1.99		*b*	Peng *et al*, EES 2016 (Ref. S4)
5.9		570		75.7	4.3		*b*	Wang *et al*, NC 2018 (Ref. S)
5	140	525	10.75	93	2.96	1.85	*bc*	Jin *et al*, ACSEL 2018 (Ref. S6)
5.4	50	493.8		236	1.0		*a*	Wei *et al*, ACSO 2019 (Ref. S7)
7.1	118	485.3		81.7	2.9		*b*	
7.2	132	493		102.8	2.4		*c*	
		506	13.18	75.9	3.37		*(100) plane*	Jin *et al*, MRB 2020 (Ref. S8)
5.1		505	12	83.3	3.1		*//bc*	Jin *et al*, JAC 2017 (Ref. S9)
		493	4.7	212.8	1.14		*⊥bc*	
2.1	361	502	11.8	84.7	2.97	2.3	*bc*	Jin *et al*, ACSAMI 2019 (Ref. S10)
4.4	34.9	504	2.56	390.6	0.65		*a* ($Sn_{1-x}Se$)	
4.4	287.5	476	20.15	49.6	4.57	1.9	*bc*($Sn_{1-x}Se$)	
11.4	153	500	11.94	83.75	3			Jin *et al*, CRT 2019 (Ref. S11)
0.058		500	9.1	109.9	2.3		*c*	Duong *et al*, NC 2016 (Ref. S12)
4.2		480	3.8	263.2	0.88		*c* (Sn_6Se)	Tang *et al*, PBCM 2019 (Ref. S13)
		932		93×10^3	0.009	1.2	*a*	Ibrahim *et al*, APL 2017 (Ref. S14)
		856		11×10^3	0.067	2.3	*b*	
		805		14×10^3	0.046	1.7	*c*	
		400		25	6.5			Nishimura *et al*, PRL 2019 (Ref. S15)
		398		21	7.5			
		396		18	8.7			

		394		15.5	10				
		391		14	11				
		390		13	12				
		474	10	100	2.3				
		464	12.5	80	2.7				
		448	17	58.8	3.4				
		441	20.5	48.8	3.9				Su *et al*, RSCA 19 (Ref. S16)
		435	25.5	39.2	4.7				
		426	31.5	31.8	5.75				
		415	38	26.3	6.65				

***p*-doped SnSe single crystals**

carrier concentration (10^{19} cm^{-3})	carrier mobility (cm^2 V^{-1} s^{-1})	Thermoelectric power (μV K^{-1})	Electrical conductivity (S cm^{-1})	Electrical resistivity (mΩ cm)	Power factor (μW K^{-2} cm^{-1})	Lattice thermal conductivity (W K^{-1} m^{-1})	Dopant or chemical composition	along axis	Reference
0.39	119.9	308.2		13.3	7.1	2.1	1% Ag	*b*	Wang *et al*, APL 2018 (Ref. S3)
1.8	94.1	262.6		3.7	18.6	1.8	1% Na	*b*	
5.2	84.4	147		1.4	15.4	1.7	2% Na	*b*	
0.27	142.9	284	61.5	16.3	4.96		1% Ag	*b*	Peng *et al*, EES 2016 (Ref. S4)
0.72	139.8	237	161	6.2	9		2% Ag	*b*	
1.27	125.5	233	255	3.9	13.8		3% Ag	*b*	
2.26	134.4	212	486	2.1	21.8		1% Na	*b*	
4.77	96.3	172	735	1.36	21.7		2% Na	*b*	
8.22	82.9	160	1090	0.92	27.9		3% Na	*b*	
4.9		160	370	2.7	9.5	0.45	1.5 % Na	*a*	Zhao *et al*, S 2016 (Ref. S17), Zhao *et al*, EES 2016 (Ref. S18)
4.9		160	1570	0.64	40.2	0.73	1.5 % Na	*b*	
4.9		160	1100	0.91	28.2	0.6	1.5 % Na	*c*	
		272	190	5.3	14.1		3% Ag	*a*	Jin *et al*, JCG 2017 (Ref. S19)
1.2	58.5	223	115	8.7	5.7	0.9		*a*	Chang *et al*, S 2018

1.2	151	231	290	3.4	15.5			*b*	(Ref. S20)
0.048	208	520	15.63	64	4.2	1.6	$SnSe_{0.9}S_{0.1}$	*b*	Peng *et al*, MT 2018 (Ref. S21)
2.5	131.4	190	514.63	1.94	18.6	1.4	$Sn_{0.99}Na_{0.01}Se_{0.9}S_{0.1}$	*b*	
4.5	124.6	158	881.32	1.13	22	1.2	$Sn_{0.98}Na_{0.02}Se_{0.9}S_{0.1}$	*b*	
9	126.8	145.5	1994	0.5	42.2	0.98	$Sn_{0.97}Na_{0.03}Se_{0.9}S_{0.1}$	*b*	
5.07	157	192.9	1273.6	0.79	47.4		$Sn_{0.985}Na_{0.015}Se+SnSe_2$		Qin *et al*, JACS 2020 (Ref. S22)
6.29	159.7	183.5	1606.8	0.62	54.1		$Sn_{0.985}Na_{0.015}Se+2\%SnSe_2$		
6.45	141.9	183	1464.8	0.68	49		$Sn_{0.985}Na_{0.015}Se+3\%SnSe_2$		
6.55	125.6	180.5	1316.1	0.76	42.9		$Sn_{0.985}Na_{0.015}Se+5\%SnSe_2$		
2.16	289	195	998.9	1	38		$Sn_{0.98}Na_{0.02}Se$		Qin *et al*, JACS 2019 (Ref. S23)
2.48	279.3	178	1108.1	0.9	35.1		$Sn_{0.98}Na_{0.02}Se_{0.995}Te_{0.005}$		
3.11	276.3	175	1374.6	0.73	42.1		$Sn_{0.98}Na_{0.02}Se_{0.99}Te_{0.01}$		
3.65	272.9	170	1593.7	0.63	46.1		$Sn_{0.98}Na_{0.02}Se_{0.985}Te_{0.015}$		
4.77	262.1	166	1999.9	0.5	55.1		$Sn_{0.98}Na_{0.02}Se_{0.98}Te_{0.02}$		
3.03	273.9	174	1328	0.75	40.2		$Sn_{0.98}Na_{0.02}Se_{0.975}Te_{0.025}$		
2.86	166.7	188	762.8	1.3	27		$Sn_{0.98}Na_{0.02}Se_{0.97}Te_{0.03}$		
		200	850	1.18	33.2		Na		Su *et al*, RSCA 19 (Ref. S16)
		199	930	1.08	36.7				
		160	1650	0.61	42.7				
		134	2450	0.41	43.9				

***n*-doped SnSe single crystals**

carrier concentration (10^{18} cm^{-3})	carrier mobility (cm^2 V^{-1} s^{-1})	Thermoelectric power (μV K^{-1})	Electrical conductivity (S cm^{-1})	Electrical resistivity (mΩ cm)	Power factor (μW K^{-2} cm^{-1})	Lattice thermal conductivity (W K^{-1} m^{-1})	Dopant or chemical composition	along axis	Reference
8.1		-233	151	6.6	8.2	0.71	Br	*a*	Chang *et al*, S 2018 (Ref. S20)
8.3		-227.5	169	5.9	8.75	0.85		*a*	
9.7		-209	178	5.6	7.78	0.81		*a*	
9.9		-208	226	4.4	9.78	0.69		*a*	
9.9		-199	231	4.3	9.15	0.78		*a*	

12	136	-181	232	4.3	7.6	0.77		*a*	
12	177	-195	342	2.9	13.0			*b*	
5.9×10^{-3}		-535	0.58	1724.1	0.17		2% Bi	*c*	
1.8×10^{-2}		-692	2.24	446.4	1.1		4% Bi	*c*	
4×10^{-2}		-650	7.7	129.9	3.25		6% Bi	*c*	Duong *et al*, NC 2016 (Ref. S12)
4×10^{-2}		-629	3.3	303.0	1.31		6% Bi	*a*	
4×10^{-2}		-545	4.59	217.9	1.36		6% Bi	*b*	
4×10^{-2}		-601	7.4	135.1	2.67		6% Bi	*c*	
0.33	45	633	2.3	434.8	0.92		$Sn_{5.85}Pb_{0.15}Se$	*c*	
0.34	58.8	-627	3.1	322.6	1.2		$Sn_{5.7}Pb_{0.3}Se$	*c*	Tang *et al*, PBCM 2019 (Ref. S13)
0.38	73	-507	4.3	232.6	1.1		$Sn_{5.4}Pb_{0.6}Se$	*c*	
0.41	77.8	-455	5	200	1.04		$Sn_{5.1}Pb_{0.9}Se$	*c*	

Undoped SnSe polycrystals

carrier concentration (10^{18} cm^{-3})	carrier mobility (cm^2 V^{-1} s^{-1})	Thermoelectric power (μV K^{-1})	Electrical conductivity (S cm^{-1})	Electrical resistivity (mΩ cm)	Power factor (μW K^{-2} cm^{-1})	Lattice thermal conductivity (W K^{-1} m^{-1})	Chemical composition	Direction	Reference
0.2	160.7	504.6	4.7	212.77	1.2				Shi *et al*, CS 2018 (Ref. S24)
2.79	39	310	17.4	57.5	1.7				Shi *et al*, AEM 2019 (Ref. S25)
4.6	59.4	264	44.5	22.5	3.1				Hong *et al*, JMCA 2017 (Ref. S26)
0.4		357.7	8.8	113.6	1.1				Chandra *et al*, JACS 2019 (Ref. S27); Chandra *et al*, ACSEM 2020 (Ref. S28)
		312.3	5.2	192.3	0.5				
0.4		465.6		534.6	0.4				Sassi *et al*, MTP 2015 (Ref. S29)
0.4		483.8		1116.9	0.2				
13.5	7.57	227	16.0	62.5	0.8				Li *et al*, IC 2018 (Ref. S30)

2.44	2.66	374	1.04	961.5	0.15				Wang *et al*, JMCC 2017 (Ref. S31)
2.53	2.59	371	1.05	952.4	0.14				
2.69	6.75	333	2.91	343.6	0.32				
2.66	6.79	348	2.89	346	0.35				
2.66	8.30	361	3.53	283.3	0.46				
0.7	1.39	200		350	0.11				Yang *et al*, JEM 2017 (Ref. S32)
6.28×10^{-3}	0.96	300	9.71×10^{-4}	1.03×10^{6}	8.74×10^{-5}			perpendicular to the pressing direction of SPS	Chen *et al*, AFM 2016 (Ref. S33)
7.46×10^{-4}	2.09	585	2.49×10^{-4}	4×10^{6}	8.52×10^{-5}				
5.4×10^{-2}	1.42	500	1.23×10^{-2}	8.13×10^{4}	3.1×10^{-3}				
0.293	49.37	546.9	2.31	432.9	0.7	1.47		perpendicular to the hot-pressing direction	Fu *et al*, JEM 2017 (Ref. S34)
0.51	166.56	485	13.4	74.6	3.15		$Sn_{0.98}Se$	$ZM_{//}$	Fu *et al*, JMCC 2016 (Ref. S35)
		508.6	0.97	1030.9	0.25		$Sn_{0.98}Se$	$ZM_{\perp}$	
0.53	19.92	487	1.74	574.7	0.41		$Sn_{1.03}Se$	ZM-SPS-$1_{//}$	
0.17	12.81	496	0.37	2702.7	0.09		$Sn_{1.02}Se$	ZM-SPS-$1_{//}$	
0.23		590	0.57	1770	0.2			along the hot-pressing direction	Zhang *et al*, AEM 2015 (Ref. S36)
0.25	45	523		637	0.43				Chen *et al*, JMCA 2014 (Ref. S37)
		500	0.033	30303	0.0083				Nguyen *et al*, NRL 2018 (Ref. S38)
		366.4	0.012	81566	0.0016				
0.225	12.5	577.8	1.14	877.2	0.83				Chang *et al*, RSCA 2016 (Ref. S39)
		577.8	2.2	454.5	1.16				
		503.7		1087.6	0.23				Sassi *et al*, APL 2014 (Ref. S40)
		482.3		456.2	0.51				
6.5	32.2	311.4	6	166.7	0.58	1.11	SnSe		Wei *et al*, JACS 2018 (Ref. S41)
10.6	4.0	304.2	6.6	151.5	0.61	1.06	$Sn_{0.95}Se$		
10.8	2.9	295.3	4.7	212.8	0.41	1.05	$Sn_{0.925}Se$		
9.8	3.4	284.9	5.2	192.3	0.42	1.04	$Sn_{0.895}Se$		

p-doped SnSe polycrystals									
carrier concentration (10^{18} cm^{-3})	carrier mobility (cm^2 V^{-1} s^{-1})	Thermoelectric power (μV K^{-1})	Electrical conductivity (S cm^{-1})	Electrical resistivity (mΩ cm)	Power factor (μW K^{-2} cm^{-1})	Lattice thermal conductivity (W K^{-1} m^{-1})	Dopant or chemical composition	Direction	Reference
0.3	112.9	481.8	5.1	196.1	1.2		$Sn_{0.99}Cu_{0.01}Se$		Shi *et al*, CS 2018 (Ref. S24)
0.5	85.6	458.4	6.4	156.3	1.3		$Sn_{0.98}Cu_{0.02}Se$		
0.9	76.2	408.1	11.6	86.2	1.9		$Sn_{0.95}Cu_{0.05}Se$		
1.7	71.6	355.3	19.5	51.3	2.5		$Sn_{0.925}Cu_{0.07}Se$		
2.9	58.3	297.9	27.4	36.5	2.4		$Sn_{0.9}Cu_{0.1}Se$		
3.4	57.2	282.7	31.6	31.6	2.5		$Sn_{0.882}Cu_{0.11}Se$		
7.3	37.8	262.2	44.2	22.6	3.0		$Sn_{0.984}Cd_{0.005}Se$		Shi *et al*, AEM 2019 (Ref. S25)
9.98	37.6	241.6	60.1	16.6	3.5		$Sn_{0.976}Cd_{0.009}Se$		
13.3	37	223	78.8	12.7	3.9		$Sn_{0.966}Cd_{0.014}Se$		
17.8	35.9	207.5	98.3	10.2	4.2		$Sn_{0.957}Cd_{0.019}Se$		
23.3	35.5	197.1	132.9	7.5	5.2		$Sn_{0.948}Cd_{0.023}Se$		
7.2	55.7	250	63.8	15.7	4		$SnSe_{0.95}Te_{0.05}$		Hong *et al*, JMCA 2017 (Ref. S26)
9.8	51.6	230.5	81.4	12.3	4.3		$SnSe_{0.9}Te_{0.1}$		
16	35.7	225	92.7	10.8	4.7		$SnSe_{0.8}Te_{0.2}$		
6.7		323.4	9.6	104.2	1		$Sn_{0.99}Ge_{0.01}Se$		Chandra *et al*, JACS 2019 (Ref. S27); Chandra *et al*, ACSEM 2020 (Ref. S28)
6.7		290.2	3.8	263.2	0.32		$Sn_{0.99}Ge_{0.01}Se$		
9		288.3	10.7	93.5	0.89		$Sn_{0.98}Ge_{0.02}Se$		
9		279.5	6.9	144.9	0.54		$Sn_{0.98}Ge_{0.02}Se$		
42		267.9	13.6	73.5	0.98		$Sn_{0.97}Ge_{0.03}Se$		
42		257.4	8.2	122	0.54		$Sn_{0.97}Ge_{0.03}Se$		
11.0	26.2	235	45.9	21.8	2.53		1% Ag		Wang *et al*, JMCC 2017 (Ref. S31)
11.6	13.6	227	25.1	39.8	1.29		2% Ag		
11.8	13.8	232	26.1	38.3	1.40		2% Ag		
10.8	33.8	224	58.3	17.2	2.93		2% Ag		
10.4	32.5	231	54.2	18.5	2.89		2% Ag		
10.4	32.9	227	53.2	18.8	2.74		2% Ag		

11.6	35.7	222	66.1	15.1	3.26		3% Ag		
4.7	8.2	270		290	0.25		2% Na		Yang *et al*, JEM 2017 (Ref. S32)
5.4	5.9	450		292	0.69		4% Na		
5.7	6.4	330		330	0.33		10% Na		
8.5	2.25	260	3.07	325.7	0.21		1% K	perpendicular to the pressing direction of SPS	Chen *et al*, AFM 2016 (Ref. S33)
9.2	2.55	232	3.76	266	0.21		1% K		
8.2	2.34	180	3.21	311.5	0.1		1% K		
0.336	37.64	526.97	2.02	495.1	0.56	1.39	4% Ge	perpendicular to the hot-pressing direction	Fu *et al*, JEM 2017 (Ref. S34)
0.308	27.9	537.10	1.37	729.9	0.4	1.26	6% Ge		
0.066	36.73	607.36	0.39	2564.1	0.14	1.06	8% Ge		
0.291	19.67	506.76	1.23	813	0.32	0.78	20% S		
3.6	46	318		51.8	1.95		1% Ag		Chen *et al*, JMCA 2014 (Ref. S37)
5.2	44.2	286		21	3.9		3% Ag		
8.9	37.4	222		7.6	6.5		5% Ag		
9.7	34.2	197		12.3	3.2		7% Ag		
18.1		144.6	15.2	65.8	0.32		1% Na		Chere *et al*, JMCA 2016 (Ref. S42)
21		203	33.3	30	1.37		1.5% Na		
26.7		139	17.7	56.5	0.34		2% Na		
		149	10.5	95.2	0.23		3% Na		
		463.4		1862	0.1				Guo *et al*, NE 2017 (Ref. S43)
		456.7		426.6	0.5				
		504.8		7321.7	0.03				
		517.6		1786	0.15		$SnSe_{0.98}Te_{0.02}$		Zhang *et al*, JAC 2016 (Ref. S44)
		383.2		326.3	0.45		$SnSe_{0.98}Te_{0.02}$		
		359.2		234.6	0.55		$SnSe_{0.98}Te_{0.02}$		
		350.5		159.5	0.77		$SnSe_{0.98}Te_{0.02}$		
		332.4		107.3	1.03		$SnSe_{0.98}Te_{0.02}$		
		289.1		101.9	0.82		$SnSe_{0.98}Te_{0.02}$		
		255.8		99.1	0.66		$SnSe_{0.98}Te_{0.02}$		
		484.2		2605	0.09		$SnSe_{0.97}Te_{0.03}$		
		431.7		810.3	0.23		$SnSe_{0.97}Te_{0.03}$		

		396		261.4	0.6		$SnSe_{0.97}Te_{0.03}$	
		373.8		194	0.72		$SnSe_{0.97}Te_{0.03}$	
		371		174.2	0.79		$SnSe_{0.97}Te_{0.03}$	
		315.4		134.4	0.74		$SnSe_{0.97}Te_{0.03}$	
		280.4		112.3	0.7		$SnSe_{0.97}Te_{0.03}$	
		505.8		3654.8	0.07		$SnSe_{0.95}Te_{0.05}$	
		460.3		1059.4	0.2		$SnSe_{0.95}Te_{0.05}$	
		449.8		843	0.24		$SnSe_{0.95}Te_{0.05}$	
		386.7		249.2	0.6		$SnSe_{0.95}Te_{0.05}$	
		380.8		245.8	0.59		$SnSe_{0.95}Te_{0.05}$	
		320.7		205.7	0.5		$SnSe_{0.95}Te_{0.05}$	
		309		194.9	0.49		$SnSe_{0.95}Te_{0.05}$	
43.1	1.7	157.9	8.7	114.9	0.22	0.97	$Na_{0.01}(Sn_{0.95}Pb_{0.05})_{0.99}Se$	Lee *et al*, J 2019 (Ref. S45)
26.8	7.5	166.5	32.5	30.8	0.9	0.69		
12.8	3.9	231.9	9.1	109.9	0.49	0.84		

***n*-doped SnSe polycrystals**

carrier concentration (10^{18} cm^{-3})	carrier mobility (cm^2 V^{-1} s^{-1})	Thermoelectric power (μV K^{-1})	Electrical conductivity (S cm^{-1})	Electrical resistivity (mΩ cm)	Power factor (μW K^{-2} cm^{-1})	Dopant or chemical composition	Reference
5×10^{-3}		-36.9	2×10^{-4}	5×10^{6}	2.7×10^{-7}	$SnSe_{0.99}I_{0.01}$	Zhang *et al*, AEM 2015 (Ref. S36)
1.5×10^{-2}		-212	2.3×10^{-3}	4.3×10^{5}	1.0×10^{-4}	$SnSe_{0.98}I_{0.02}$	
4.6×10^{-2}		-243	0.1	8×10^{4}	7.4×10^{-3}	$SnSe_{0.97}I_{0.03}$	
0.44		-215	0.6	1.76×10^{3}	2.6×10^{-2}	$SnSe_{0.96}I_{0.04}$	
3.8×10^{-2}		-206	0.2	6.3×10^{3}	6.7×10^{-3}	$SnSe_{0.87}S_{0.1}I_{0.03}$	
2.5×10^{-2}		-238	0.07	1.4×10^{4}	4×10^{-3}	$SnSe_{0.67}S_{0.3}I_{0.03}$	

4.8×10^{-2}	0.43	-254.4	3×10^{-4}	3×10^{6}	2.0×10^{-5}	$SnSe_{0.99}Br_{0.01}$	Chang *et al*, RSCA 2016 (Ref. S39)
5.9×10^{-2}	0.87	-222.7	0.05	2.1×10^{4}	2.33×10^{-3}	$SnSe_{0.98}Br_{0.02}$	
1.83	0.6	-232.2	0.2	5.1×10^{3}	1.1×10^{-2}	$SnSe_{0.97}Br_{0.03}$	
10.2	0.32	-194	0.3	3.5×10^{3}	1.1×10^{-2}	$SnSe_{0.96}Br_{0.04}$	
3.31	0.6	-207	0.05	1.9×10^{4}	2.3×10^{-3}	$Sn_{0.975}Pb_{0.025}Se_{0.97}Br_{0.03}$	
0.66	0.14	-194	0.03	3.6×10^{4}	1.1×10^{-3}	$Sn_{0.95}Pb_{0.05}Se_{0.97}Br_{0.03}$	
0.21	1.56	-169	0.07	1.3×10^{4}	2.1×10^{-3}	$Sn_{0.9}Pb_{0.1}Se_{0.97}Br_{0.03}$	
5.48	0.63	-167	0.46	2.2×10^{3}	1.3×10^{-2}	$Sn_{0.8}Pb_{0.2}Se_{0.97}Br_{0.03}$	
12.3	1.84	-112	2.6	384.6	3.3×10^{-2}	$Sn_{0.7}Pb_{0.3}Se_{0.97}Br_{0.03}$	
0.24	1.2	-160	0.46	2173.9	0.01	$SnSe_{0.95}$	Wang *et al*, APL 2016 (Ref. S46)
15.6	4	-179	10.1	99.2	0.32	$SnSe_{0.95}$-0.1$BiCl_3$	
12.7	5.9	-179	11.8	85.0	0.38	$SnSe_{0.95}$-0.2$BiCl_3$	
10.7	10.2	-168	17.4	57.4	0.49	$SnSe_{0.95}$-0.4$BiCl_3$	
7.8	9.6	-196	11.8	84.7	0.45	$SnSe_{0.95}$-0.6$BiCl_3$	
2.25×10^{-3}	35.57	-587.9	0.01	1.0×10^{5}	3.5×10^{-3}	$SnSb_{0.01}Se_{0.98}$	Shi *et al*, AEM 2018 (Ref. S47)
0.15	4.02	-377.1	0.1	1.0×10^{4}	0.01	$SnSb_{0.02}Se_{0.96}$	
6.1	0.65	-198.5	0.81	1.2×10^{3}	0.03	$SnSb_{0.03}Se_{0.94}$	
		-237	0.02	5.39×10^{4}	1.04×10^{-3}	2% Bi	Nguyen *et al*, NRL 2018 (Ref. S38)
		-286.4	0.02	6.57×10^{4}	1.25×10^{-3}	2% Bi	
		-54.6	0.36	2.77×10^{3}	1.08×10^{-3}	4% Bi	
		-121.5	0.24	4.23×10^{3}	3.49×10^{-3}	4% Bi	
		-52.7	0.07	1.53×10^{4}	1.81×10^{-4}	6% Bi	
		-103.8	0.14	6.92×10^{3}	1.56×10^{-3}	6% Bi	
		-116.5	0.19	5.36×10^{3}	2.54×10^{-3}	8% Bi	
		-59.5	0.18	5.42×10^{3}	6.54×10^{-3}	8% Bi	
4.7×10^{-4}	13.91	-893	1.06×10^{-3}	9.43×10^{5}	8.45×10^{-4}	$Sn_{0.99}Bi_{0.01}Se$	Li *et al*, IC 2018 (Ref. S30)
8.5×10^{-4}	11.62	-839	1.58×10^{-3}	6.33×10^{5}	1.11×10^{-3}	$Sn_{0.98}Bi_{0.02}Se$	
1.3×10^{-3}	18.78	-791	4.07×10^{-3}	2.46×10^{5}	2.55×10^{-3}	$Sn_{0.97}Bi_{0.03}Se$	
1.7×10^{-3}	7.22	-724	1.95×10^{-3}	5.13×10^{5}	1.02×10^{-3}	$Sn_{0.96}Bi_{0.04}Se$	

Nanocrystalline SnSe samples							
carrier concentration (10^{18} cm^{-3})	carrier mobility (cm^2 V^{-1} s^{-1})	Thermoelectric power (μV K^{-1})	Electrical conductivity (S cm^{-1})	Electrical resistivity (mΩ cm)	Power factor (μW K^{-2} cm^{-1})	Crystalline state / dopant	Reference
4.91	8.9	560		140.4	2.23	Porous SnSe Nanosheets	Ju *et al*, CM 2017 (Ref. S48)
		503<br>855.5<br>900<br>465<br>516.8		6300<br>53×10^4<br>81×10^4<br>72×10^4<br>25×10^4	0.04<br>1.4×10^{-3}<br>1.0×10^{-3}<br>3×10^{-4}<br>1.1×10^{-3}	nanocrystalline Ge-doped SnSe	Gharsallah *et al*, SR 2016 (Ref. S49)
7.95×10^{-3}		501<br>557		6400<br>6800	0.04<br>0.05	Nanocrystalline SnSe	Serrano-Sánchez *et al*, APL 2015 (Ref. S50)
0.5<br>1.2<br>5.2<br>9	139<br>67<br>18<br>13	198<br>182<br>162<br>123	11.12<br>12.84<br>14.62<br>19.03	89.9<br>77.9<br>68.4<br>52.5	0.43<br>0.43<br>0.38<br>0.28	Ag doped SnSe nanocrystals	Chien *et al*, RSCA 2017 (Ref. S51)
		379.5<br>137.7<br>51.81<br>25.5<br>-35.8<br>-49.8<br>-98.9<br>-118<br>-125<br>-181<br>-270.5<br>-370<br>-430		36005<br>9480<br>5835<br>6503<br>2101<br>1494<br>978<br>819<br>977<br>1310<br>563<br>1141<br>2641	4×10^{-3}<br>2×10^{-3}<br>4.6×10^{-4}<br>1×10^{-4}<br>6.1×10^{-4}<br>1.7×10^{-3}<br>0.01<br>0.02<br>0.02<br>0.03<br>0.13<br>0.12<br>0.07	nanocrystalline SnSe thin films	Shimizu *et al*, SR 2021 (Ref. S52)
		326<br>304	6.3<br>4.2	158.7<br>238.1	0.67<br>0.39	phase-separated nanostructured<br>$Sn_{0.98}Pb_{0.01}Zn_{0.01}Se$<br>$Sn_{0.97}Pb_{0.01}Zn_{0.02}Se$	Liu *et al*, NE 2018 (Ref. S53)

229	11.7	85.5	0.61	$Sn_{0.96}Pb_{0.01}Zn_{0.03}Se$

Table S2. The power factor, PF and the figure of merit, *ZT* data for selected thermoelectrics at 300 K or close temperatures.

Compound	Power factor ($\mu W\ K^{-2}\ cm^{-1}$)	*ZT*	Reference
$(Bi_{1-x}Sb_x)_2Te_3$ alloys	~30-60	1.1-1.8	Fan *et al*, APL 10 (Ref. S54),<br>Poudel *et al*, S 2008 (Ref. S55),<br>Kim *et al*, S 2015 (Ref. S56),<br>Soleimani *et al*, SETA 2020 (Ref. S57),<br>Yu *et al*, GGGEGR 2020 (Ref. S58)
AgCuTe ($(AgCu)_{1-x}Te_{0.9}Se_{0.1}$)	12	0.7	Jiang *et al*, JMCA 2020 (Ref. S59)
n-type Mg_3Sb ($Mg_{3.2-y}A_ySb_{1.5}Bi_{0.49}Se_{0.01}$, *A*=Mn, Co)	12-21	0.5-0.65	Zhang, F. *et al*, AFM 2020 (Ref. S60)
n-type Te-doped Mg_3Sb_2 single crystals ($Mg_{3+\delta}Sb_{2-x}Te_x$)	23	0.4	Imasato *et al*, AM 2020 (Ref. S61)
n-type Mg_3Sb_2 ($Mg_3Sb_{1.5-0.5x}Bi_{0.5-0.5x}Te_x$)	7-14	0.3-0.56	Zhang *et al*, NC 2017 (Ref. S62)
n-type Mg_3Bi_2-based alloys	20-25	0.7	Mao *et al*, S 2019 (Ref. S63)
MgAgSb ($Mg_{1-x}Li_xAg_{0.97}Sb_{0.9}$)	15-24	0.4-0.8	Liu *et al.*, AEM 2016 (Ref. S64)
Nanostructured α-MgAgSb ($MgAg_{0.97}Sb_{0.99-x}B_x$)	15.5-19	0.5-0.7	Gao *et al*, JMCC 2018 (Ref. S65)
MgAgSb	10.5-15	0.8-1.1	Zheng *et al*, NE 2019 (Ref. S66)
Nanostructured $Mg_{1-x}Na_xAg_{0.97}Sb_{0.99}$	19-21	0.7-0.8	Shuai *et al*, NE 2015 (Ref. S67)
Nanostructured monoclinic Cu_2Se (at 380 K)	6.7	0.72	Chen *et al*, N 2020 (Ref. S68)
β-Ag_2Se	25	0.7	Yang *et al*, JMCA 2017 (Ref. S69)
$Cd_{1-x}Ag_xSb$	15-19	0.5	Wang *et al*, CM 2015 (Ref. S70)
Cd-doped polycrystalline $AgSbTe_2$	13.6	1.5	Roychowdhury *et al*, S 2021 (Ref. S71)
Bi-doped PbTe	37	0.48	Lee *et al*, ACSN 2019 (Ref. S72)
$(GeTe)_x(AgSbTe_2)_{100-x}$ compounds	21	0.55	Yang *et al*, N 2008 (Ref. S73)
$Ag_8Sn_{1.005}Se_6$ (at 327 K)	6.15	0.65	Wang *et al*, CEC 20 (Ref. S74)
Stress-released orthorhombic phase of SnTe	80		Morozova *et al*, APL 2021 (Ref. S75)
p-type half-Heusler alloys, $Nb_{1-x}Ti_xFeSb$	106	0.25	He *et al*, PNAS 2016 (Ref. S76)
F4-TCNQ doped $FASnI_3$ thin films	130	0.19	Zheng *et al*, JMCA 2020 (Ref. S77)
One-dimensional telluride, Ta_4SiTe_4	170		Inohara *et al*, APL 2017 (Ref. S78)
Ultrathin FeSe film	260	1.5	Shimizu *et al*, NC 2019 (Ref. S79)

References

[S1] R. Kilaas, *J. Microsc.* **1998**, *190*, 45.

[S2] L.-D. Zhao, S.-H. Lo, Y. Zhang, H. Sun, G. Tan, C. Uher, C. Wolverton, V. P. Dravid, M. G. Kanatzidis, *Nature* **2014**, *508*, 373.

[S3] S. Wang, S. Hui, K. Peng, T. P. Bailey, W. Liu, Y. Yan, X. Zhou, X. Tang, C. Uher, *Appl. Phys. Lett.* **2018**, *112*, 142102.

[S4] K. Peng, X. Lu, H. Zhan, S. Hui, X. Tang, G. Wang, J. Dai, C. Uher, G. Wang, X. Zhou, *Energy Environ. Sci.* **2016**, *9*, 454.

[S5] Z. Wang, C. Fan, Z. Shen, C. Hua, Q. Hu, F. Sheng, Y. Lu, H. Fang, Z. Qiu, J. Lu, Z. Liu, W. Liu, Y. Huang, Z.-A. Xu, D. W. Shen, Y. Zheng, *Nat. Commun.* **2018**, *9*, 47.

[S6] M. Jin, Z. Chen, X. Tan, H. Shao, G. Liu, H. Hu, J. Xu, B. Yu, H. Shen, J. Xu, H. Jiang, Y. Pei, J. Jiang, *ACS Energy Lett.* **2018**, *3*, 689.

[S7] P.-C. Wei, S. Bhattacharya, Y.-F. Liu, F. Liu, J. He, Y.-H. Tung, C.-C. Yang, C.-R. Hsing, D.-L. Nguyen, C.-M. Wei, M.-Y. Chou, Y.-C. Lai, T.-L. Hung, S.-Y. Guan, C.-S. Chang, H.-J. Wu, C.-H. Lee, W.-H. Li, R. P. Hermann, Y.-Y. Chen, A. M. Rao, *ACS Omega* **2019**, *4*, 5442.

[S8] M. Jin, Z. Tang, J. Jiang, R. Zhang, L. Zhou, S. Zhao, Y. Chen, Y. Chen, X. Wang, R. Li, *Mater. Res. Bull.* **2020**, *126*, 110819.

[S9] M. Jin, H. Shao, H. Hu, D. Li, H. Shen, J. Xu, J. Jiang, *J. Alloys Compd.* **2017**, *712*, 857.

[S10] M. Jin, X.-L. Shi, T. Feng, W. Liu, H. Feng, S. T. Pantelides, J. Jiang, Y. Chen, Y. Du, J. Zou, Z.-G. Chen, *ACS Appl. Mater. Interfaces* **2019**, *11*, 8051.

[S11] M. Jin, J. Jiang, R. Li, X. Wang, Y. Chen, Y. Chen, J. Xu, *Cryst. Res. Technol.* **2019**, *54*, 1900032.

[S12] A. T. Duong, V. Q. Nguyen, G. Duvjir, V. T. Duong, S. Kwon, J. Y. Song, J. K. Lee, J. E. Lee, S. Park, T. Min, J. Lee, J. Kim, S. Cho, *Nat. Commun.* **2016**, *7*, 13713.

[S13] Y. Tang, L. Shen, Z. Chen, L. Sun, W. Liu, J. Liu, S. Deng, *Phys. B Condens. Matter* **2019**, *570*, 128.

[S14] D. Ibrahim, J.-B. Vaney, S. Sassi, C. Candolfi, V. Ohorodniichuk, P. Levinsky, C. Semprimoschnig, A. Dauscher, B. Lenoir, *Appl. Phys. Lett.* **2017**, *110*, 032103.

[S15] T. Nishimura, H. Sakai, H. Mori, K. Akiba, H. Usui, M. Ochi, K. Kuroki, A. Miyake, M. Tokunaga, Y. Uwatoko, K. Katayama, H. Murakawa, N. Hanasaki, *Phys. Rev. Lett.* **2019**, *122*, 226601.

[S16] N. Su, B. C. Qin, K. J. Zhu, Z. Y. Liu, P. Shahi, J. P. Sun, B. S. Wang, Y. Sui, Y. G. Shi, L. D. Zhao, J.-G. Cheng, *RSC Adv.* **2019**, *9*, 26831.

[S17] L.-D. Zhao, G. Tan, S. Hao, J. He, Y. Pei, H. Chi, H. Wang, S. Gong, H. Xu, V. P. Dravid, C. Uher, G. J. Snyder, C. Wolverton, M. G. Kanatzidis, *Science* **2016**, *351*, 141.

[S18] L.-D. Zhao, C. Chang, G. Tan, M. G. Kanatzidis, *Energy Environ. Sci.* **2016**, *9*, 3044.

[S19] M. Jin, H. Shao, H. Hu, D. Li, J. Xu, G. Liu, H. Shen, J. Xu, H. Jiang, J. Jiang, *J. Cryst. Growth* **2017**, *460*, 112.

[S20] C. Chang, M. Wu, D. He, Y. Pei, C.-F. Wu, X. Wu, H. Yu, F. Zhu, K. Wang, Y. Chen, L. Huang, J.-F. Li, J. He, L.-D. Zhao, *Science* **2018**, *360*, 778.

[S21] K. Peng, B. Zhang, H. Wu, X. Cao, A. Li, D. Yang, X. Lu, G. Wang, X. Han, C. Uher, X. Zhou, *Mater. Today* **2018**, *21*, 501.

[S22] B. Qin, Y. Zhang, D. Wang, Q. Zhao, B. Gu, H. Wu, H. Zhang, B. Ye, S. J. Pennycook, L.-D. Zhao, *J. Am. Chem. Soc.* **2020**, *142*, 5901.

[S23] B. Qin, D. Wang, W. He, Y. Zhang, H. Wu, S. J. Pennycook, L.-D. Zhao, *J. Am. Chem. Soc.* **2019**, *141*, 1141.

[S24] X. Shi, K. Zheng, M. Hong, W. Liu, R. Moshwan, Y. Wang, X. Qu, Z.-G. Chen, J. Zou, *Chem. Sci.* **2018**, *9*, 7376.

[S25] X. Shi, A. Wu, T. Feng, K. Zheng, W. Liu, Q. Sun, M. Hong, S. T. Pantelides, Z.-G. Chen, J. Zou, *Adv. Energy Mater.* **2019**, *9*, 1803242.

[S26] M. Hong, Z.-G. Chen, L. Yang, T. C. Chasapis, S. D. Kang, Y. Zou, G. J. Auchterlonie, M. G. Kanatzidis, G. J. Snyder, J. Zou, *J. Mater. Chem. A* **2017**, *5*, 10713.

[S27] S. Chandra, K. Biswas, *J. Am. Chem. Soc.* **2019**, *141*, 6141.

[S28] S. Chandra, P. Dutta, K. Biswas, *ACS Appl. Energy Mater.* **2020**, *3*, 9051.

[S29] S. Sassi, C. Candolfi, J.-B. Vaney, V. Ohorodniichuk, P. Masschelein, A. Dauscher, B. Lenoir, *Mater. Today Proc.* **2015**, *2*, 690.

[S30] X. Li, C. Chen, W. Xue, S. Li, F. Cao, Y. Chen, J. He, J. Sui, X. Liu, Y. Wang, Q. Zhang, *Inorg. Chem.* **2018**, *57*, 13800.

[S31] S. Wang, S. Hui, K. Peng, T. P. Bailey, X. Zhou, X. Tang, C. Uher, *J. Mater. Chem. C* **2017**, *5*, 10191.

[S32] Z.-R. Yang, W.-H. Chen, C.-J. Liu, *J. Electron. Mater.* **2017**, *46*, 2964.

[S33] Y.-X. Chen, Z.-H. Ge, M. Yin, D. Feng, X.-Q. Huang, W. Zhao, J. He, *Adv. Funct. Mater.* **2016**, *26*, 6836.

[S34] Y. Fu, J. Xu, G.-Q. Liu, X. Tan, Z. Liu, X. Wang, H. Shao, H. Jiang, B. Liang, J. Jiang, *J. Electron. Mater.* **2017**, *46*, 3182.

[S35] Y. Fu, J. Xu, G.-Q. Liu, J. Yang, X. Tan, Z. Liu, H. Qin, H. Shao, H. Jiang, B. Liang, J. Jiang, *J. Mater. Chem. C* **2016**, *4*, 1201.

[S36] Q. Zhang, E. K. Chere, J. Sun, F. Cao, K. Dahal, S. Chen, G. Chen, Z. Ren, *Adv. Energy Mater.* **2015**, *5*, 1500360.

[S37] C.-L. Chen, H. Wang, Y.-Y. Chen, T. Day, G. J. Snyder, *J. Mater. Chem. A* **2014**, *2*, 11171.

[S38] V. Q. Nguyen, T. H. Nguyen, V. T. Duong, J. E. Lee, S.-D. Park, J. Y. Song, H.-M. Park, A. T. Duong, S. Cho, *Nanoscale Res. Lett.* **2018**, *13*, 200.

[S39] C. Chang, Q. Tan, Y. Pei, Y. Xiao, X. Zhang, Y.-X. Chen, L. Zheng, S. Gong, J.-F. Li, J. He, L.-D. Zhao, *RSC Adv.* **2016**, *6*, 98216.

[S40] S. Sassi, C. Candolfi, J.-B. Vaney, V. Ohorodniichuk, P. Masschelein, A. Dauscher, B. Lenoir, *Appl. Phys. Lett.* **2014**, *104*, 212105.

[S41] W. Wei, C. Chang, T. Yang, J. Liu, H. Tang, J. Zhang, Y. Li, F. Xu, Z. Zhang, J.-F. Li, G. Tang, *J. Am. Chem. Soc.* **2018**, *140*, 499.

[S42] E. K. Chere, Q. Zhang, K. Dahal, F. Cao, J. Mao, Z. Ren, *J. Mater. Chem. A* **2016**, *4*, 1848.

[S43] J. Guo, J. Jian, J. Liu, B. Cao, R. Lei, Z. Zhang, B. Song, H. Zhao, *Nano Energy* **2017**, *38*, 569.

[S44] Y. Zhang, X. Jia, H. Sun, B. Sun, B. Liu, H. Liu, L. Kong, H. Ma, *J. Alloys Compd.* **2016**, *667*, 123.

[S45] Y. K. Lee, Z. Luo, S. P. Cho, M. G. Kanatzidis, I. Chung, *Joule* **2019**, *3*, 719.

[S46] X. Wang, J. Xu, G. Liu, Y. Fu, Z. Liu, X. Tan, H. Shao, H. Jiang, T. Tan, J. Jiang, *Appl. Phys. Lett.* **2016**, *108*, 083902.

[S47] X.-L. Shi, K. Zheng, W.-D. Liu, Y. Wang, Y.-Z. Yang, Z.-G. Chen, J. Zou, *Adv. Energy Mater.* **2018**, *8*, 1800775.

[S48] H. Ju, M. Kim, D. Park, J. Kim, *Chem. Mater.* **2017**, *29*, 3228.

[S49] M. Gharsallah, F. Serrano-Sánchez, N. M. Nemes, F. J. Mompeán, J. L. Martínez, M. T. Fernández-Díaz, F. Elhalouani, J. A. Alonso, *Sci. Rep.* **2016**, *6*, 26774.

[S50] F. Serrano-Sánchez, M. Gharsallah, N. M. Nemes, F. J. Mompean, J. L. Martínez, J. A. Alonso, *Appl. Phys. Lett.* **2015**, *106*, 083902.

[S51] C.-H. Chien, C.-C. Chang, C.-L. Chen, C.-M. Tseng, Y.-R. Wu, M.-K. Wu, C.-H. Lee, Y.-Y. Chen, *RSC Adv.* **2017**, *7*, 34300.

[S52] S. Shimizu, K. Miwa, T. Kobayashi, Y. Tazawa, S. Ono, *Sci. Rep.* **2021**, *11*, 1637.

[S53] J. Liu, P. Wang, M. Wang, R. Xu, J. Zhang, J. Liu, D. Li, N. Liang, Y. Du, G. Chen, G. Tang, *Nano Energy* **2018**, *53*, 683.

[S54] S. Fan, J. Zhao, J. Guo, Q. Yan, J. Ma, H. H. Hng, *Appl. Phys. Lett.* **2010**, *96*, 182104.

[S55] B. Poudel, Q. Hao, Y. Ma, Y. Lan, A. Minnich, B. Yu, X. Yan, D. Wang, A. Muto, D. Vashaee, X. Chen, J. Liu, M. S. Dresselhaus, G. Chen, Z. Ren, *Science* **2008**, *320*, 634.

[S56] S. Il Kim, K. H. Lee, H. A. Mun, H. S. Kim, S. W. Hwang, J. W. Roh, D. J. Yang, W. H. Shin, X. S. Li, Y. H. Lee, G. J. Snyder, S. W. Kim, *Science* **2015**, *348*, 109.

[S57] Z. Soleimani, S. Zoras, B. Ceranic, S. Shahzad, Y. Cui, *Sustain. Energy Technol. Assessments* **2020**, *37*, 100604.

[S58] K. Yu, Y. Zhou, Y. Liu, F. Liu, L. Hu, W. Ao, C. Zhang, Y. Li, J. Li, H. Xie, *Geomech. Geophys. Geo-Energy Geo-Resources* **2020**, *6*, 12.

[S59] J. Jiang, H. Zhu, Y. Niu, Q. Zhu, S. Song, T. Zhou, C. Wang, Z. Ren, *J. Mater. Chem. A* **2020**, *8*, 4790.

[S60] F. Zhang, C. Chen, H. Yao, F. Bai, L. Yin, X. Li, S. Li, W. Xue, Y. Wang, F. Cao, X. Liu, J. Sui, Q. Zhang, *Adv. Funct. Mater.* **2020**, *30*, 1906143.

[S61] K. Imasato, C. Fu, Y. Pan, M. Wood, J. J. Kuo, C. Felser, G. J. Snyder, *Adv. Mater.* **2020**, *32*, 1908218.

[S62] J. Zhang, L. Song, S. H. Pedersen, H. Yin, L. T. Hung, B. B. Iversen, *Nat. Commun.* **2017**, *8*, 13901.

[S63] J. Mao, H. Zhu, Z. Ding, Z. Liu, G. A. Gamage, G. Chen, Z. Ren, *Science* **2019**, *365*, 495.

[S64] Z. Liu, Y. Wang, J. Mao, H. Geng, J. Shuai, Y. Wang, R. He, W. Cai, J. Sui, Z. Ren, *Adv. Energy Mater.* **2016**, *6*, 1502269.

[S65] W. Gao, X. Yi, B. Cui, Z. Wang, J. Huang, J. Sui, Z. Liu, *J. Mater. Chem. C* **2018**, *6*, 9821.

[S66] Y. Zheng, C. Liu, L. Miao, C. Li, R. Huang, J. Gao, X. Wang, J. Chen, Y. Zhou, E. Nishibori, *Nano Energy* **2019**, *59*, 311.

[S67] J. Shuai, H. S. Kim, Y. Lan, S. Chen, Y. Liu, H. Zhao, J. Sui, Z. Ren, *Nano Energy* **2015**, *11*, 640.

[S68] J. Chen, T. Liu, D. Bao, B. Zhang, G. Han, C. Liu, J. Tang, D. Zhou, L. Yang, Z.-G. Chen, *Nanoscale* **2020**, *12*, 20536.

[S69] D. Yang, X. Su, F. Meng, S. Wang, Y. Yan, J. Yang, J. He, Q. Zhang, C. Uher, M. G. Kanatzidis, X. Tang, *J. Mater. Chem. A* **2017**, *5*, 23243.

[S70] S. Wang, J. Yang, L. Wu, P. Wei, J. Yang, W. Zhang, Y. Grin, *Chem. Mater.* **2015**, *27*, 1071.

[S71] S. Roychowdhury, T. Ghosh, R. Arora, M. Samanta, L. Xie, N. K. Singh, A. Soni, J. He, U. V. Waghmare, K. Biswas, *Science* **2021**, *371*, 722.

[S72] M. H. Lee, J. H. Yun, G. Kim, J. E. Lee, S.-D. Park, H. Reith, G. Schierning, K. Nielsch, W. Ko, A.-P. Li, J.-S. Rhyee, *ACS Nano* **2019**, *13*, 3806.

[S73] S. H. Yang, T. J. Zhu, T. Sun, J. He, S. N. Zhang, X. B. Zhao, *Nanotechnology* **2008**, *19*, 245707.

[S74] X. Wang, C. Liu, J. Chen, L. Miao, S. Wu, X. Wang, Z. Xie, W. Xu, Q. Chen, *CrystEngComm* **2020**, *22*, 248.

[S75] N. V. Morozova, I. V. Korobeynikov, S. V. Ovsyannikov, *Appl. Phys. Lett.* **2021**, *118*, 103903.

[S76] R. He, D. Kraemer, J. Mao, L. Zeng, Q. Jie, Y. Lan, C. Li, J. Shuai, H. S. Kim, Y. Liu, D. Broido, C.-W. Chu, G. Chen, Z. Ren, *Proc. Natl. Acad. Sci.* **2016**, *113*, 13576.

[S77] L. Zheng, T. Zhu, Y. Li, H. Wu, C. Yi, J. Zhu, X. Gong, *J. Mater. Chem. A* **2020**, *8*, 25431.

[S78] T. Inohara, Y. Okamoto, Y. Yamakawa, A. Yamakage, K. Takenaka, *Appl. Phys. Lett.* **2017**, *110*, 183901.

[S79] S. Shimizu, J. Shiogai, N. Takemori, S. Sakai, H. Ikeda, R. Arita, T. Nojima, A. Tsukazaki, Y. Iwasa, *Nat. Commun.* **2019**, *10*, 825.
